# Large-scale GWAS identifies multiple loci for hand grip strength providing biological insights into muscular fitness

**DOI:** 10.1038/ncomms16015

**Published:** 2017-07-12

**Authors:** Sara M. Willems, Daniel J. Wright, Felix R. Day, Katerina Trajanoska, Peter K. Joshi, John A. Morris, Amy M. Matteini, Fleur C. Garton, Niels Grarup, Nikolay Oskolkov, Anbupalam Thalamuthu, Massimo Mangino, Jun Liu, Ayse Demirkan, Monkol Lek, Liwen Xu, Guan Wang, Christopher Oldmeadow, Kyle J. Gaulton, Luca A. Lotta, Eri Miyamoto-Mikami, Manuel A. Rivas, Tom White, Po-Ru Loh, Mette Aadahl, Najaf Amin, John R. Attia, Krista Austin, Beben Benyamin, Søren Brage, Yu-Ching Cheng, Paweł Cięszczyk, Wim Derave, Karl-Fredrik Eriksson, Nir Eynon, Allan Linneberg, Alejandro Lucia, Myosotis Massidda, Braxton D. Mitchell, Motohiko Miyachi, Haruka Murakami, Sandosh Padmanabhan, Ashutosh Pandey, Ioannis Papadimitriou, Deepak K. Rajpal, Craig Sale, Theresia M. Schnurr, Francesco Sessa, Nick Shrine, Martin D. Tobin, Ian Varley, Louise V. Wain, Naomi R. Wray, Cecilia M. Lindgren, Daniel G. MacArthur, Dawn M. Waterworth, Mark I. McCarthy, Oluf Pedersen, Kay-Tee Khaw, Douglas P. Kiel, GEFOS Any-Type of Fracture Consortium, Ling Oei, Hou-Feng Zheng, Vincenzo Forgetta, Aaron Leong, Omar S. Ahmad, Charles Laurin, Lauren E. Mokry, Stephanie Ross, Cathy E. Elks, Jack Bowden, Nicole M. Warrington, Anna Murray, Katherine S. Ruth, Konstantinos K. Tsilidis, Carolina Medina-Gómez, Karol Estrada, Joshua C. Bis, Daniel I. Chasman, Serkalem Demissie, Anke W. Enneman, Yi-Hsiang Hsu, Thorvaldur Ingvarsson, Mika Kähönen, Candace Kammerer, Andrea Z. Lacroix, Guo Li, Ching-Ti Liu, Yongmei Liu, Mattias Lorentzon, Reedik Mägi, Evelin Mihailov, Lili Milani, Alireza Moayyeri, Carrie M. Nielson, Pack Chung Sham, Kristin Siggeirsdotir, Gunnar Sigurdsson, Kari Stefansson, Stella Trompet, Gudmar Thorleifsson, Liesbeth Vandenput, Nathalie van der Velde, Jorma Viikari, Su-Mei Xiao, Jing Hua Zhao, Daniel S. Evans, Steven R. Cummings, Jane Cauley, Emma L. Duncan, Lisette C. P. G. M. de Groot, Tonu Esko, Vilmundar Gudnason, Tamara B. Harris, Rebecca D. Jackson, J Wouter Jukema, Arfan M. A. Ikram, David Karasik, Stephen Kaptoge, Annie Wai Chee Kung, Terho Lehtimäki, Leo-Pekka Lyytikäinen, Paul Lips, Robert Luben, Andres Metspalu, Joyce B. J. van Meurs, Ryan L. Minster, Erick Orwoll, Edwin Oei, Bruce M. Psaty, Olli T. Raitakari, Stuart W. Ralston, Paul M. Ridker, John A. Robbins, Albert V. Smith, Unnur Styrkarsdottir, Gregory J. Tranah, Unnur Thorstensdottir, Andre G. Uitterlinden, Joseph Zmuda, M Carola Zillikens, Evangelia E. Ntzani, Evangelos Evangelou, John P. A. Ioannidis, David M. Evans, Claes Ohlsson, Yannis Pitsiladis, Noriyuki Fuku, Paul W. Franks, Kathryn N. North, Cornelia M. van Duijn, Karen A. Mather, Torben Hansen, Ola Hansson, Tim Spector, Joanne M. Murabito, J. Brent Richards, Fernando Rivadeneira, Claudia Langenberg, John R. B. Perry, Nick J. Wareham, Robert A. Scott

**Affiliations:** 1 MRC Epidemiology Unit, University of Cambridge School of Clinical Medicine, Cambridge CB2 0QQ, UK; 2 Department of Internal Medicine, Erasmus Medical Center, 3015 CE Rotterdam, The Netherlands; 3 Department of Epidemiology, Erasmus Medical Center, 3015 CE Rotterdam, The Netherlands; 4 Usher Institute for Population Health Sciences and Informatics, University of Edinburgh, Edinburgh EH8 9AB, UK; 5 Centre for Clinical Epidemiology, Lady Davis Institute for Medical Research, Jewish General Hospital, McGill University, Montreal, Quebec, Canada QC H3T 1E2; 6 Department of Human Genetics, McGill University, Montreal, Quebec, Canada H3G 0B1; 7 Division of Geriatric Medicine and Gerontology, Johns Hopkins University School of Medicine, Baltimore, Maryland 21287, USA; 8 Queensland Brain Institute, University of Queensland, St Lucia, Queensland 4072, Australia; 9 The Novo Nordisk Foundation Center for Basic Metabolic Research, Faculty of Health and Medical Sciences, University of Copenhagen, 2200 Copenhagen, Denmark; 10 Lund University Diabetes Center, Department of Clinical Sciences, Diabetes and Endocrinology, Skånes University Hospital, 222 41 Lund, Sweden; 11 Centre for Healthy Brain Ageing, School of Psychiatry, University of New South Wales, Sydney, New South Wales 2031, Australia; 12 Department of Twin Research & Genetic Epidemiology, Kings College London, London SE1 7EH, UK; 13 NIHR Biomedical Research Centre at Guy’s and St. Thomas’ NHS Foundation Trust, London SE1 9RT, UK; 14 Department of Human Genetics, Leiden University Medical Center, 2333 ZA Leiden, The Netherlands; 15 Analytic and Translational Genetics Unit, Massachusetts General Hospital, Boston, Maryland 02114, USA; 16 Harvard Medical School, Boston, Maryland 02115, USA; 17 Centre for Sport and Exercise Science and Medicine (SESAME), University of Brighton, Eastbourne BN20 7SN, UK; 18 Hunter Medical Research Institute, Newcastle, New South Wales 2305, Australia; 19 Department of Pediatrics, University of California San Diego, La Jolla, California 92093, USA; 20 Japan Society for the Promotion of Science, Tokyo 102-0083, Japan; 21 Department of Sports and Life Science, National Institute of Fitness and Sports, Kanoya, Kagoshima 891-2393, Japan; 22 Department of Biomedical Data Sciences, Stanford University, Stanford, California 94305, USA; 23 BROAD Institute of the Massachusetts Institute of Technology and Harvard University, Cambridge, Massachusetts 02142, USA; 24 Department of Epidemiology, Harvard T.H. Chan School of Public Health, Boston, Massachusetts 02115, USA; 25 Research Centre for Prevention and Health, Capital Region of Denmark, Glostrup University Hospital, DK-2600 Glostrup, Denmark; 26 Faculty of Health and Medicine, University of Newcastle, Newcastle, New South Wales 2308, Australia; 27 John Hunter Hospital, New Lambton, New South Wales 2305, Australia; 28 Institute for Molecular Bioscience, University of Queensland, St Lucia, Queensland 4072, Australia; 29 Division of Endocrinology Diabetes and Nutrition, Department of Medicine, University of Maryland School of Medicine, Baltimore, Maryland 21201, USA; 30 Faculty of Physical Education, Gdańsk University of Physical Education and Sport, 80-336 Gdańsk, Poland; 31 Department of Movement and Sports Sciences, Ghent University, 9000 Ghent, Belgium; 32 Institute of Sport, Exercise & Active Living (ISEAL), Victoria University, Melbourne, Victoria 8001, Australia; 33 Murdoch Children’s Research Institute, Royal Children’s Hospital, Melbourne, Victoria 3052, Australia; 34 Department of Clinical Experimental Research, Rigshospitalet, 2600 Glostrup, Denmark; 35 Department of Clinical Medicine, Faculty of Health and Medical Sciences, University of Copenhagen, DK-2200 Copenhagen, Denmark; 36 Universidad Europea de Madrid, 28670 Villaviciosa de Odón, Madrid, Spain; 37 Research Institute ‘i+12’, Hospital Universitario 12 de Octubre, 28041 Madrid, Spain; 38 Department of Life and Environmental Sciences, University of Cagliari, 09124 Cagliari, Italy; 39 Geriatrics Research and Education Clinical Center, Baltimore Veterans Administration Medical Center, Baltimore, Maryland 21201, USA; 40 National Institutes of Biomedical Innovation, Health and Nutrition, Tokyo 162-8636, Japan; 41 British Heart Foundation Glasgow Cardiovascular Research Centre, Institute of Cardiovascular and Medical Sciences, College of Medical, Veterinary and Life Sciences, University of Glasgow, Glasgow G12 8QQ, UK; 42 Target Sciences, GlaxoSmithKline, King of Prussia, Pennsylvania 19406, USA; 43 Musculoskeletal Physiology Research Group, Sport, Health and Performance Enhancement (SHAPE) Research Centre, Nottingham Trent University, Nottingham NG1 4FQ, UK; 44 Department of Clinical and Experimental Medicine, Medical Genetics, University of Foggia, 71122 Foggia FG, Italy; 45 Department of Health Sciences, University of Leicester, Leicester LE1 7RH, UK; 46 National Institute for Health Research, Leicester Respiratory Biomedical Research Unit, Glenfield Hospital, Leicester LE3 9QP, UK; 47 The Big Data Institute, University of Oxford, Oxford OX3 7BN, UK; 48 Wellcome Trust Centre for Human Genetics, Nuffield Department of Medicine, University of Oxford, Oxford OX3 7BN, UK; 49 Oxford Centre for Diabetes, Endocrinology and Metabolism, University of Oxford, Oxford OX3 7LE, UK; 50 NIHR Oxford Biomedical Research Centre, Oxford OX3 7LE, UK; 51 Department of Public Health and Primary Care, University of Cambridge, Cambridge CB2 0SR, UK; 52 Institute for Aging Research, Hebrew SeniorLife, Boston, Massachusetts 02131, USA; 53 Department of Medicine, Beth Israel Deaconess Medical Centre, Boston, Massachusetts 02215, USA; 54 Graduate School of Health and Sports Science, Juntendo University, Chiba 270-1695, Japan; 55 Genetic and Molecular Epidemiology Unit, Department of Clinical Sciences, Lund University, Skånes University Hospital, 222 41 Lund, Sweden; 56 Public Health and Clinical Medicine, Section for Medicine, Umeå University, 901 87 Umeå, Sweden; 57 Biobank Research, Umeå University, 901 87 Umeå, Sweden; 58 Faculty of Health Sciences, University of Southern Denmark, 5230 Odense M, Denmark; 59 Boston University School of Medicine, Department of Medicine, Section of General Internal Medicine, Boston, Massachusetts 02118, USA; 60 National Heart Lung and Blood Institute’s and Boston University’s Framingham Heart Study, Framingham, Massachusetts 01702, USA; 61 Department of Medicine, McGill University, Montreal, Quebec, Canada H3G 1A4; 62 Institute of Aging Research, School of Medicine, Hangzhou Normal University, 311121 Hangzhou, Zhejiang, China; 63 Affiliated Hospital of Hangzhou Normal University, 310000 Hangzhou, Zhejiang, China; 64 Lady Davis Institute, Jewish General Hospital, McGill University, Montréal, Quebec, Canada H3T 1E2; 65 Division of General Internal Medicine, Massachusetts General Hospital, Boston, Massachusetts 02114, USA; 66 Department of Medicine, Harvard Medical School, Boston, Massachusetts 02115, USA; 67 MRC Integrative Epidemiology Unit, University of Bristol, Bristol BS8 2BN, UK; 68 Departments of Clinical Epidemiology and Biostatistics, McMaster University, Hamilton, Ontario, Canada L8S 4L8; 69 Personalised Healthcare & Biomarkers, Innovative Medicines and Early Development Biotech Unit, AstraZeneca, Cambridge CB4 0FZ, UK; 70 The University of Queensland Diamantina Institute, Translational Research Institute, Brisbane, Queensland 4102, Australia; 71 Genetics of Complex Traits, University of Exeter Medical School, University of Exeter, Exeter EX1 2LU, UK; 72 Department of Hygiene and Epidemiology, University of Ioannina Medical School, Mpizani 455 00, Greece; 73 Department of Epidemiology and Biostatistics, Imperial College London, Faculty of Medicine, School of Public Health, London SW7 2AZ, UK; 74 Cardiovascular Health Research Unit, Department of Medicine, University of Washington, Seattle, Washington 98101, USA; 75 Division of Preventive Medicine, Brigham and Women's Hospital, Boston, Massachusetts 02115, USA; 76 Department of Biostatistics, Boston University School of Public Health, Boston, Massachusetts 02118, USA; 77 Molecular and Integrative Physiological Sciences, Harvard TH Chan School of Public Health, Boston, Massachusetts 02115, USA; 78 Department of Orthopedic Surgery, Akureyri Hospital, 600 Akureyri, Iceland; 79 Institution of Health Science, University of Akureyri, 600 Akureyri, Iceland; 80 Department of Clinical Physiology, Tampere University Hospital, 33521 Tampere, Finland; 81 Department of Clinical Physiology, University of Tampere School of Medicine, 33014 Tampere, Finland; 82 Department of Human Genetics, Graduate School of Public Health, University of Pittsburgh, Pittsburgh, Pennsylvania 15261, USA; 83 Women’s Health Center of Excellence, Family Medicine and Public Health, University of California San Diego, La Jolla, California 92093, USA; 84 Division of Public Health Science, Wake Forest University School of Medicine, Winston-Salem, North Carolina 27101, USA; 85 Centre for Bone and Arthritis Research, Department of Internal Medicine and Geriatric Medicine, Institute of Medicine, Sahlgrenska Academy, University of Gothenburg, 413 90 Gothenburg, Sweden; 86 Estonian Genome Center, University of Tartu, 51010 Tartu, Estonia; 87 School of Public Health, Oregon Health & Science University, Portland, Oregon 97239, USA; 88 Department of Psychiatry, The University of Hong Kong, Hong Kong 999077, China; 89 Icelandic Heart Association, 201 Kopavogur, Iceland; 90 Department of Endocrinology and Metabolism, Landspitali, The National University Hospital of Iceland, 101 Reykjavík, Iceland; 91 Faculty of Medicine, University of Iceland, 101 Reykjavík, Iceland; 92 deCODE Genetics/Amgen, 101 Reykjavík, Iceland; 93 Department of Cardiology, Leiden University Medical Center, 2333 ZA Leiden, The Netherlands; 94 Department of Internal Medicine, Academic Medical Center, 1105 AZ Amsterdam, The Netherlands; 95 Division of Medicine, Turku University Hospital, 20521 Turku, Finland; 96 Department of Medicine, University of Turku, 20521 Turku, Finland; 97 Department of Medicine, The University of Hong Kong, Hong Kong 999077, China; 98 California Pacific Medical Center, Research Institute, San Francisco, California 94114, USA; 99 Department of Epidemiology, Graduate School of Public Health, University of Pittsburgh, Pittsburgh, Pennsylvania 15261, USA; 100 Department of Human Nutrition, Wageningen University, 6708 PB Wageningen, The Netherlands; 101 Laboratory of Epidemiology, National Institute on Aging, National Institutes of Health, Bethesda, Maryland 20892, USA; 102 Department of Medicine, Division of Endocrinology, Diabetes and Metabolism, The Ohio State University, Columbus, Ohio 43210, USA; 103 Department of Radiology, Erasmus Medical Center, 3015 CE Rotterdam, The Netherlands; 104 Department of Neurology, Erasmus Medical Center, 3015 CE Rotterdam, The Netherlands; 105 Faculty of Medicine in the Galilee, Bar-Ilan University, Safed 5290002, Israel; 106 Fimlab Laboratories, Department of Clinical Chemistry, 33520 Tampere, Finland; 107 Department of Clinical Chemistry, University of Tampere School of Medicine, 33014 Tampere, Finland; 108 Department of Internal Medicine, VU University Hospital, 1081 HV Amsterdam, The Netherlands; 109 Institute of Molecular and Cell Biology, University Of Tartu, 51010 Tartu, Estonia; 110 Bone and Mineral Research Unit, Department of Medicine, Oregon Health & Science University, Portland, Oregon 97201, USA; 111 Group Health Research Institute, Group Health Cooperative, Seattle, Washington 98101, USA; 112 Department of Clinical Physiology and Nuclear Medicine, Turku University Hospital, 20521 Turku, Finland; 113 Research Centre of Applied and Preventive Cardiovascular Medicine, University of Turku, 20520 Turku, Finland; 114 Institute of Genetics and Molecular Medicine, University of Edinburgh, Western General Hospital, Edinburgh EH4 2XU, UK; 115 Department of Medicine, University of California, Davis, Sacramento, California 95616, USA; 116 Netherlands Genomics Initiative (NGI)-sponsored Netherlands Consortium for Healthy Aging (CHA), Leiden 2300 RC, The Netherlands; 117 Center for Evidence-based Medicine, Brown University School of Public Health, Providence, Rhode Island 02912, USA; 118 Stanford Prevention Research Center, Stanford University, Palo Alto, California 94305, USA

## Abstract

Hand grip strength is a widely used proxy of muscular fitness, a marker of frailty, and predictor of a range of morbidities and all-cause mortality. To investigate the genetic determinants of variation in grip strength, we perform a large-scale genetic discovery analysis in a combined sample of 195,180 individuals and identify 16 loci associated with grip strength (*P*<5 × 10^−8^) in combined analyses. A number of these loci contain genes implicated in structure and function of skeletal muscle fibres (*ACTG1*), neuronal maintenance and signal transduction (*PEX14, TGFA, SYT1*), or monogenic syndromes with involvement of psychomotor impairment (*PEX14, LRPPRC* and *KANSL1*). Mendelian randomization analyses are consistent with a causal effect of higher genetically predicted grip strength on lower fracture risk. In conclusion, our findings provide new biological insight into the mechanistic underpinnings of grip strength and the causal role of muscular strength in age-related morbidities and mortality.

## Abstract (web summary)

Hand grip strength as a proxy of muscular fitness is a clinical predictor of mortality and morbidity. In a large-scale GWA study, the authors find 16 robustly associated genetic loci that highlight roles in muscle fibre structure and function, neuronal maintenance and nervous system signal transduction.

Muscle strength, measured by isometric hand grip strength, is an accessible and widely used proxy of muscular fitness. Lower grip strength is associated with impaired quality of life in older adults, and is an established marker of frailty, predicting physical decline and functional limitation in daily living^1^,^2^,^3^. The value of grip strength as a clinical predictor of fracture risk has been demonstrated in different populations^4^,^5^, and higher grip strength has been found to be prognostic of walking recovery after hip fracture surgery in later life^6^. Grip strength has also been shown to predict cardiovascular disease (CVD) and all-cause mortality over many years of follow-up^7^,^8^,^9^. Whilst it remains unclear whether these prospective associations with fracture risk, CVD and mortality are causal—or reflect early manifestation of underlying disease processes—the role of muscular strength as a predictor of functional capacity highlights the importance of understanding its aetiology.

Grip strength is highly heritable (*h*^2^=30–65%)^10^,^11^,^12^. Whilst candidate gene approaches have implicated multiple loci in this phenotype, including thermogenic and myogenic factors^13^,^14^, there remain few robustly replicated associations. Two genome-wide association studies in up to 27,000 individuals have been reported to date^15^,^16^, yielding one intergenic genome-wide significant association^16^.

Here, in a combined sample size of 195,180 individuals, including 142,035 individuals from the UK Biobank (UKB) cohort^17^, we identified 16 genome-wide significant loci associated with grip strength. We also performed Mendelian randomization (MR) analyses, which showed no evidence for causality in the associations of grip strength with CVD or all-cause mortality, but were suggestive of a causal effect of muscular strength on fracture risk.

## Results

### Multiple novel loci are associated with grip strength

**Figure 1:**
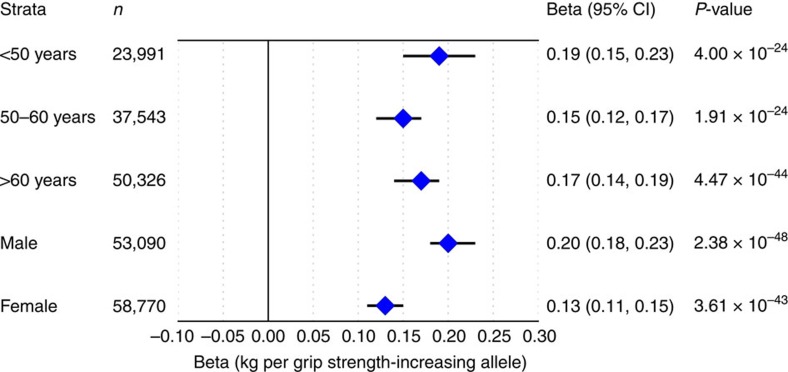
Association of the 16 SNV grip strength score with grip strength by age and sex strata. Association of the grip strength-increasing genetic score showed no interaction with observed grip strength by age (*p*_interaction_=0.30) but was stronger in men than in women (*P*_interaction_=1.56 × 10^−5^) in a subset of 111,860 unrelated UK Biobank participants from stage one analyses. Associations shown are from linear regression.

In stage one analyses, we tested the association of >17 million variants (minor allele frequency (MAF)>0.1%, imputation quality >0.4), in 142,035 white European individuals from UK Biobank (Supplementary Table 1) with maximal grip strength. Genome-wide single-nucleotide variant (SNV) heritability was estimated at 23.9% (SE 2.7%). Twenty-one loci showed genome-wide significant associations (*P*<5 × 10^−8^) in stage one (Supplementary Fig. 1), and were subsequently followed up in stage two analyses of up to 53,145 individuals from 8 additional studies (Supplementary Table 1; Supplementary Note) including the Cohorts for Heart and Aging Research in Genomic Epidemiology (CHARGE) consortium^16^. Twelve loci were independently replicated (directional consistency with stage one, *P<*0.05) in stage two cohorts (Supplementary Table 2A) and 16 loci contained genome-wide significant associations (*P*<5 × 10^−8^) in combined analyses. Effect sizes on grip strength ranged from 0.14 to 0.42 kg per allele under an additive model (Table 1; Supplementary Fig. 1; Supplementary Table 2A and B). Given the discordance in sample size between stage one and two analyses, and in the interests of maximizing power, we considered there to be evidence of association at any locus reaching genome-wide significance in combined analyses, and pursued all 16 in downstream analyses. Lead SNVs at the 16 grip strength-associated loci included common variants (MAF≥5%) in or near *POLD3, TGFA, ERP27, HOXB3, GLIS1, PEX14, MGMT, LRPPRC, SYT1, GBF1, KANSL1, SLC8A1, IGSF9B, ACTG1,* a low-frequency variant (MAF 3%) in *DEC1,* and a further common variant falling within the human leukocyte antigen (HLA) region (Table 1; Supplementary Fig. 2). Approximate conditional analyses identified no additional signals at genome-wide significance at these 16 loci after conditioning on their respective lead SNVs. At two loci, we saw evidence for a departure from additivity (*P*<3.13 × 10^−3^ under a dominance deviation model (see Methods)); at the *GBF1* locus, we saw evidence for a dominant effect of the grip strength-raising A allele (*P*_domdev_=2.3 × 10^−3^; Supplementary Fig. 3A), and at the *SYT1* locus, we saw evidence for a recessive effect of the grip strength-raising A allele (*P*_domdev_=3.0 × 10^−3^; Supplementary Fig. 3B). No individual variants showed significant effect modification by age or sex (Supplementary Table 2C and D). The association of the 16 SNV genetic score (modelled as the sum of the grip strength-increasing allele dosage at each SNV per individual) showed no interaction with age (*P*_interaction_=0.30), but was stronger in men than in women (men: *β*=0.20 kg per grip strength-increasing allele, *P*=2.38 × 10^−48^; women: *β*=0.13 kg per grip strength-increasing allele, *P*=3.61 × 10^−43^; *P*_interaction_=1.56 × 10^−5^; Fig. 1; Supplementary Table 2C and D). Age at recruitment was independent of strength-increasing allele dosage at each of the 16 SNVs from combined analyses. Equally, allele frequency at each SNP was not predicted by age, suggesting that there is no selection of alleles by age at these loci^18^. We did not replicate the previously-reported association at rs752045 with grip strength^16^ (*β* per minor allele (95% confidence interval (CI))=0.01 (−0.06, 0.08), *P*=0.75).

A number of associated loci contained genes with biologically plausible roles in strength and neuromuscular fitness, through effects on the structure and function of skeletal muscle (*ACTG1*), excitation-contraction coupling (*SLC8A1*), evidence for neurotrophic roles *(TGFA*), or involvement in the regulation of neurotransmission (*SYT1*). *ACTG1* (Actin, γ1A) encodes a key component of the costamere—a protein complex localized to the Z-disc of skeletal muscle which physically tethers myofibrils to the cell membrane and transmits contractile force generated at the sarcomere to the extracellular matrix via the dystrophin glycoprotein complex (DGC)^19^,^20^. Monogenic loss of elements of the DGC results in muscular dystrophies^21^, whilst *Actg1* knockout mice display overt muscle weakness, progressive myopathy and decreased isometric twitch force^22^. *SLC8A1* encodes a transmembrane Na^+^/Ca^2+^ exchanger which is vital to restoring Ca^2+^ concentration to pre-excitation levels in excitable cells. Muscle-specific overexpression of *SLC8A1* has been shown to induce dystrophy-like skeletal muscle pathology^23^. Synaptotagmin-1, encoded by *SYT1*, is an integral synaptic membrane protein which regulates Ca^2+^-dependent neurotransmitter release at the presynaptic terminal^24^, and is implicated in development of neuromuscular junction pathology in rodent models of spinal muscular atrophy^25^. *TGFA* encodes transforming growth factor alpha, a well-characterized growth factor which plays a key neurotrophic role in the central and peripheral nervous systems^26^, and is upregulated during the acute injury response of motor neurons, promoting neuronal survival^27^,^28^.

Three lead variants for grip strength map in or near genes implicated in monogenic syndromes characterized by neurological and/or psychomotor impairment (Table 1; Supplementary Fig. 2). rs10186876 (*P*_combined_=9.75 × 10^−11^) lies 15 kb upstream of *LRPPRC* (leucine-rich pentacotripeptide-containing), which has been implicated in the French-Canadian variant of Leigh Syndrome (MIM: 220111), a cytochrome C oxidase deficiency with features including developmental delay, hypotonia and weakness^29^. Mutations in *PEX14* (Peroxisomal Biogenesis Factor 14) (intronic lead variant rs6687430) underlie certain forms of Zellweger Spectrum Peroxisomal Biogenesis Disorder (MIM: 614887), a syndrome characterized by absence of functional peroxisomes and systemic neurological impairment^30^. Finally, rs80103986 is intronic in *KANSL1*, which has been implicated in the complex impaired-psychomotor phenotype of Koolen-de Vries syndrome (MIM: 610443)^31^. Further, the signal at *KANSL1* is in a large linkage disequilibrium (LD) block also containing *MAPT* (rs754512, *P*_discovery_=3.7 × 10^−8^), which encodes the microtubule-associated tau protein. *MAPT* has been implicated in a suite of so-called tauopathies characterized by progressive neurological deficit, and is also a risk locus for Parkinson’s disease^32^. 17q21.31 has a complex haplotype structure comprising an inversion and three structural copy number variants arising from duplication events, which has previously been shown to be of relevance to health^33^. After imputing the nine common structural haplotypes at this locus^33^,^34^,^35^, haplotype was significantly associated with grip strength. In particular, the inverted haplotype was associated with lower strength (*β*=−0.17 kg, *P*=3.85 × 10^−6^), independent of age, sex, height and BMI (Supplementary Table 3A). This association appeared to be driven by the inverted α2.γ2 structural variant (*β*=−0.18 kg, *P*=1.24 × 10^−5^).

In sensitivity analyses, we re-tested associations of the 16 grip strength variants in UKB after exclusion of up to 8,676 individuals with type 1 diabetes, cancer, or other prevalent disease with potential to influence muscle strength. No loci showed significant attenuation of effect relative to overall analyses (Supplementary Table 3B and C).

### Signals are enriched for biologically relevant tissues

To identify enrichment of association signals across different tissues and identify likely effector tissues, we performed cell type-specific partitioned heritability^36^ analyses on genome-wide association results from the discovery phase. After adjustment for multiple testing across nine distinct tissue types (*P*<0.0056), we observed significant enrichments of associations with grip strength in tissue-specific regulatory regions for a number of tissues, including bone/connective tissue (*P*=2.03 × 10^−10^), skeletal muscle (*P*=1.88 × 10^−9^) and the CNS (*P*=7.37 × 10^−8^). Enrichments at weaker levels of statistical significance were also observed in cardiovascular and gastrointestinal tissue, as well as the adrenal/pancreas axis, and ‘other’ tissues (Supplementary Fig. 4).

### Integration of gene expression data

Guided by tissue-specific enrichments, we sought to identify putative effector transcripts underlying these associations by investigating associations of lead SNVs or their proxies (*r*^2^>0.8) with transcript levels in brain, tibial nerve and skeletal muscle in GTEx (Supplementary Table 4). The grip strength-increasing allele at *ACTG1 (*rs6565586) was associated with lower expression of *ACTG1* in skeletal muscle (*P*_*EXP-Lead*_*=*3.81 × 10^−13^*, P*_*EXP-Best eQTL*_=2.64 × 10^−13^, *r*^2^=0.85), counter to directions suggested by *actg1* knockout in mouse. At *LRPPRC* (rs10186876), the strength-increasing allele was associated with higher *LRPPRC* expression levels in cerebellum (*P*_*EXP-Lead*_*=*6.35 × 10^−7^*, P*_*EXP-Best eQTL*_=9.35 × 10^−8^, *r*^2^=0.93) and cerebellar hemisphere (*P*_*EXP-Lead*_*=*1.29 × 10^−6^*, P*_*EXP-Best eQTL*_=3.62 × 10^−8^, *r*^2^=1.00), which appears directionally concordant with previously characterized loss of function and otherwise damaging mutations associated with the disease phenotype of French-Canadian Leigh Syndrome^37^. At rs1161433 (*ERP27* locus), the grip strength-increasing allele was associated with higher levels of *MGP* expression in tibial nerve (*P*_*EXP-Lead*_*=*5.90 × 10^−10^*, P*_*EXP-Best eQTL*_=2.19 × 10^−12^, *r*^2^=0.84). *MGP* (Matrix Gla Protein) is a well-characterized inhibitor of vascular tissue and cartilage calcification, and consequently acts as a key regulator of bone formation^38^.

We also performed integrated transcriptome-wide analyses using recently-described MetaXcan^39^ and SMR approaches^40^. Accounting for 5973 independent expression probes (Bonferroni-corrected *P*≤8.37 × 10^−6^ for *α*≤0.05), and potential coincidental overlap of eQTL signals with GWAS loci, SMR analyses using whole blood transcriptome data^41^ suggested correlation between higher grip strength and lower expression levels of *ERP27* (*P*_*SMR*_*=*2.50 × 10^−9^) and *KANSL1* (*P*_*SMR*_*=*3.05 × 10^−7^), both of which are implicated genes from our GWAS analysis.

**Figure 2:**
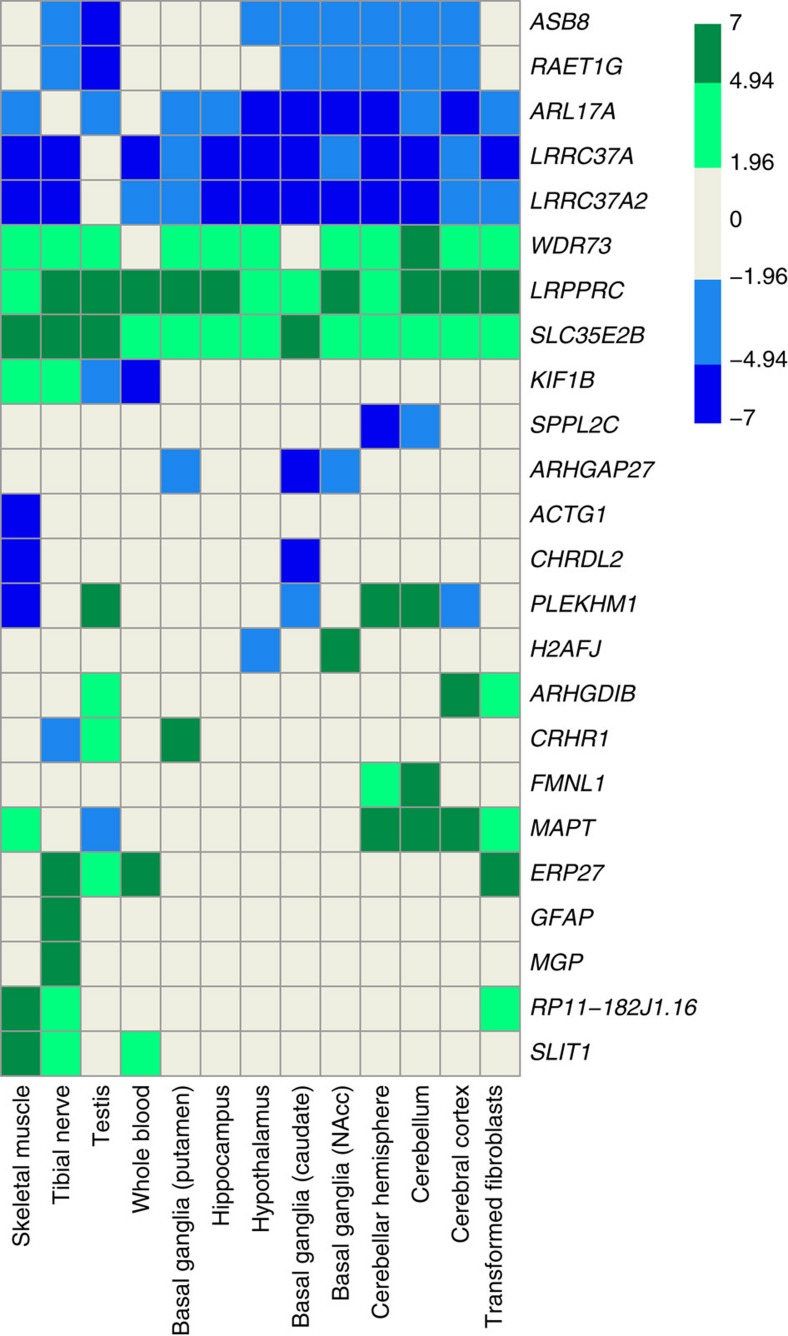
MetaXcan-predicted association of predicted gene transcript levels with grip strength across biologically relevant tissues in GTEx. Data are shown for all genes at which altered transcription was significantly associated with grip strength in at least one biologically relevant tissue, after accounting for multiple testing. Data are *z*-scores of transcript level association with higher handgrip strength, clustered by tissue. Direction of *z*-score indicates whether higher or lower gene expression is associated with higher grip strength. Absolute *z*-score>1.96 indicates nominal significance at *P*≤0.05, and ≥4.94 indicates significance after adjustment for multiple testing (*P*≤7.91 × 10^−7^). NAcc, nucleus accumbens.

MetaXcan analysis identified 25 protein-coding transcripts implicated in grip strength at Bonferroni-corrected significance in at least one of twelve biologically relevant tissues from the GTEx resource (neuronal, muscle, connective, androgenic tissues and whole blood; Supplementary Table 5). Transcripts showed concordantly altered expression across a number of these candidate tissue types (Fig. 2). For *LRPPRC,* for example, we observed association of higher expression levels across a number of brain tissue types, tibial nerve, whole blood and testis, with higher grip strength. Higher *MAPT* expression in multiple brain regions known to be implicated in motor coordination (cortex, cerebellum and cerebellar hemisphere) was also associated with higher grip strength.

### Pathways underlying variation in grip strength

Hypothesis-free gene set enrichment analysis (GSEA) based on gene-sets of common functional annotation, or belonging to pre-defined canonical pathways (Supplementary Table 6A and B), indicated five-fold enrichment of association in/near genes implicated in ‘positive regulation of protein catabolic process’ (gene ontology (GO): 1903364, false discovery rate (FDR)=0.026), and nominal enrichment of associations near genes implicated in ‘dual excision repair in global genomic nucleotide excision repair’ (Reactome: R-HSA-5696400, FDR=0.047). Given the identification of established psychomotor disease loci amongst index variants for grip strength, we additionally interrogated our association results for enrichment of genes known to be implicated in monogenic myopathies and dystrophies (Supplementary Table 6B). Grip strength associations were nominally enriched in the myopathy-linked gene set (*P*=0.017), but not at loci implicated in dystrophic conditions (*P*=0.47).

### Insights into overlap with pro-atrophic signalling

Myokine signalling via activin type II receptors (ActRII) has been recognized as a key pathway by which muscle mass might be preserved in many clinical contexts^42^,^43^; yet, we saw no evidence for enrichment of associations around genes in a custom-defined pathway of myostatin/activin signalling through ActRII (ref. 42; Supplementary Table 6A and B; described in more detail in the methods section). We also performed gene-based association analyses using VEGAS^44^ for genes encoding receptors and ligands in the ActRII signalling pathway, as well as known atrophy effectors (Supplementary Table 7). Two genes (*ACVR2B* and *FBXO32*) showed a significant association with grip strength (*P*<0.0071, accounting for seven gene-based tests; Supplementary Table 7). *ACVR2B*, for which we found the strongest evidence for gene-based association (*P*=0.0002), encodes the principal transmembrane receptor of myostatin: the target of BYM338, a monoclonal antibody-based inhibitor of ActRIIB, which has shown early promise in reversing muscle atrophy and promoting hypertrophy in phase I trials^45^,^46^. *FBXO32* encodes the E3 ubiquitin ligase Atrogin-1/MAFbx, which is recognized as fundamental effector of atrophy^42^.

### Implication of loci in elite athletic performance

Whilst poor grip strength is normally considered a marker of frailty, we also investigated the role of grip strength-associated SNVs in the opposite extreme of physiology: elite athlete status. We examined the association of grip strength-associated SNVs with odds of being an elite sprint/power athlete in a meta-analysis of four studies of sprint and power athletes (*N*_athletes_=616, *N*_controls_=1,610; see Methods and Supplementary Note for further details of methods and participants). Among the 14 available SNVs, we saw no evidence of association with elite athlete status (Supplementary Table 8). Previously, a nonsense mutation (R577X) in *ACTN3*, which encodes the actin-binding protein α-actinin 3 in skeletal muscle, has been associated with elite athlete status^47^. We found a nominally significant association of the stop-gain variant (T allele) with lower grip strength (additive: *β*=−0.062 kg, *P*=0.018) (Supplementary Table 9) although we found no evidence for any departure from an additive genetic model at this locus (*P*_domdev_*=*0.72; Supplementary Fig. 5).

### Variant association with muscle histology

We also examined whether the lead SNVs at grip strength-associated loci were associated with inverse-normalized muscle fibre type and capillary density in a small sample in which muscle fibre type histology and genome-wide genotyping were available (13 of 16 SNVs were available; Supplementary Table 10). Allowing for 13 tests (*P*<3.8 × 10^−3^), the grip strength-raising allele at *TGFA* was associated with a lower proportion of type I (slow-twitch oxidative) muscle fibres (*β*=−0.16, *P*=3.3 × 10^−3^), and a tendency towards higher proportion of type IIB (fast-twitch glycolytic) muscle fibres (*β*=0.16, *P*=5.5 × 10^−3^) (Supplementary Table 10). We acknowledge limited power in this small sample to identify modest effect sizes. Given a minor allele frequency of 0.1 and a sample size of 656 individuals, we estimated 80% power to detect an effect size of ∼0.35 SDs.

### MR of intermediate phenotypes on muscle strength

Given the roles of sex- and growth hormones and related phenotypes in muscle growth and development^48^,^49^,^50^, we performed summary statistic MR^51^,^52^ to test whether genetically-determined sex hormone binding globulin (SHBG), dehydroepiandrosterone sulphate (DHEA-S), insulin and insulin-like growth factor-I (IGF-I) levels were associated with grip strength. Using genome-wide significantly associated SNVs for SHBG (ref. 53), DHEA-S (ref. 54) and IGF-1 levels^55^, we saw no evidence for a causal association with grip strength (Supplementary Table 11). We saw some indication of causality of insulin resistance and fasting insulin levels in grip strength (Supplementary Table 11) in inverse-variance and median-weighted analyses, although the considerable heterogeneity in inverse-variance weighted results warrants a cautious interpretation (Supplementary Table 11; Supplementary Fig. 6).

### Muscle strength as a possible causal exposure

Using a MR approach, we investigated the potentially causal role of muscular strength in both mortality and disease outcomes, utilizing the 16 replicated loci as an instrumental variable to model genetically determined grip strength as a proxy of wider muscular strength.

**Figure 3:**
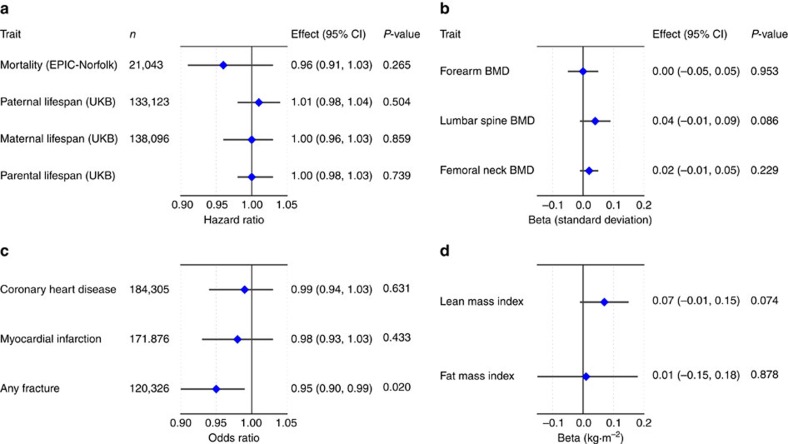
Mendelian randomization estimates of the association of grip strength with mortality and morbidity outcomes. (**a**) Mortality and parental lifespan in UKB and EPIC-Norfolk; (**b**) forearm bone mineral density (BMD), lumbar spine BMD and femoral neck BMD in GEFOS; (**c**) coronary heart disease and myocardial infarction in CARDIoGRAMplusC4D, and fracture risk in GEFOS+EPIC-Norfolk; (**d**) lean mass index and fat mass index in the Fenland Study+EPIC-Norfolk (*n*=12,851). Error bars reflect 95% CI.

Mortality: We found no evidence for a causal relationship between muscular strength and all-cause mortality in 21,043 participants (5,699 deaths) drawn from the EPIC-Norfolk cohort (hazard ratio (HR) per kg higher grip strength (95% CI): 0.96 (0.91, 1.03), *P*=0.265) (Fig. 3a). However, given wide CIs we also sought to improve power using a recently published approach leveraging data on parental lifespan^56^. Using this approach in UKB (102,072 paternal deaths, 83,315 maternal deaths), we again found no evidence for causality, with greater precision (HR (95% CI): 1.00 (0.98, 1.03), *P*=0.739) (Fig. 3a).

Coronary heart disease. We next investigated a causal role for grip strength in cardiovascular disease using genome-wide association results for coronary heart disease (CHD; 60,801 cases, 123,504 controls) and myocardial infarction (MI; 43,677 cases, 128,199 controls) from the CARDIoGRAMplusC4D Consortium^57^ (Fig. 3c). We found no evidence for a causal relationship between grip strength and CHD (odds ratio (OR) per genetically predicted kg higher grip strength (95% CI): 0.99 (0.94, 1.03), *P*=0.631) or MI (OR (95% CI): 0.98 (0.93, –1.03), *P*=0.433) (Supplementary Table 12). This result was further supported by cross-trait LD Score regression results showing no significant genetic correlation between grip strength and CHD (*r*_g_=−0.045, *P*=0.362; Supplementary Table 13).

Fracture risk and bone mineral density: We performed MR analyses of fracture risk using a meta-analysis of 1) summary statistic MR results for fracture risk from the Genetic Factors for Osteoporosis (GEFOS) consortium (*n* cases=20,439; *n* controls=78,843) (Supplementary Note; Supplementary Table 12), and 2) logistic regression results from association of the weighted grip strength genetic score with fracture risk in the EPIC-Norfolk study (1,002 cases, 20,042 controls). Meta-analysis results suggested a potential causal association of genetically-predicted higher grip strength with lower risk of fracture (OR per genetically predicted kg higher grip strength (95% CI): 0.95 (0.90–0.99), *P*=0.02; Fig. 3b). Summary statistic MR of genetically determined grip strength on publicly available bone mineral density (BMD) GWAS results^58^ did not show significant associations between grip strength and BMD (Fig. 3c; Supplementary Table 12). However, we did find genome-wide genetic correlations of bone mineral density with grip strength (femoral neck BMD: *r*_g_=0.123, *P*=9.5 × 10^−3^; lumbar spine BMD: *r*_g_=0.156, *P*=6 × 10^−4^) (Supplementary Table 13), supportive of a role for genetically predicted grip strength in fracture risk.

Similar to the BMD results, MR analyses of genetically-determined grip strength on meta-analysed GWAS results comprising 12,851 participants from the Fenland and EPIC-Norfolk cohorts did not show significant associations between grip strength and lean mass index (LMI) or fat mass index (FMI) (LMI: *β*=0.072 kg m^−2^, *P*=0.074; FMI: *β*=0.013 kg m^−2^, *P*=0.878) (Fig. 3d; Supplementary Table 12). A significant genetic correlation was observed between grip strength and LMI (*r*_g_=0.258, *P*=2.8 × 10^−5^), but not FMI (Supplementary Table 13).

## Discussion

We have identified 16 loci associated with maximal hand grip strength at genome-wide significance. A number of the lead variants were located within or close to genes implicated in structure and function of skeletal muscle fibres, neuronal maintenance and signal transduction in the central and peripheral nervous systems. Partitioned heritability analyses indicated significant tissue-specific enrichment of skeletal muscle, CNS, connective tissue and bone in the genome-wide grip strength results. We observed evidence of shared genetic aetiology between lean mass and grip strength, while pathway analyses indicated a role for genes involved in regulation of protein catabolism in the aetiology of grip strength.

Due to the well-established observational associations of grip strength with mortality and incident CHD it has been hypothesized that improvement of muscle strength might increase longevity and reduce risk of adverse cardiovascular events^7^. Our MR analyses do not find evidence supportive of a causal role of muscular strength in mortality risk, nor in risk of cardiovascular events (CHD and MI), leaving open the possibility that these observational associations may be attributable to confounding and/or reverse causality. Regardless, this does not negate the importance of maintaining strength and muscle mass during ageing as a strategy to maintain physical function^59^, and we acknowledge the potential limitations of our MR. For example, the limited variance in intermediate traits explained by genetic variants leaves uncertainty over the presence of a small causal effect. Thus, expanded genetic discovery efforts and greater availability of large-scale studies of disease outcomes will improve the precision of MR analyses in future. We saw evidence for shared genetic aetiology of bone mineral density and lean mass with grip strength, and MR results suggested a causal role for higher muscular strength in lower risk of fracture. Collectively, these results suggest that the determinants of muscular strength are shared with the determinants of fracture risk and are consistent with findings from intervention studies to increase muscle strength, which have been shown to improve functional capacity and reduce the rate of falls^59^, as well as attenuating the rate of functional decline and increased frailty which often follows major fracture among the elderly^60^. Despite the established decline in grip strength with increasing age, we did not observe heterogeneity in the effect of grip strength-associated variants with grip strength by age. However, we did observe that the cumulative effect of the genetic score was greater in men than women.

We saw evidence of enrichment of associations with grip strength around genes implicated in myopathies. We also noted three loci with genome-wide significant associations containing genes (*KANSL1, PEX14* and *LRPPRC*) implicated in rare, severe clinical syndromes characterized by phenotypes of progressive psychomotor impairment, muscle hypotonia and neuropathy^29^,^31^. Within the UKB discovery cohort, the association of all three loci with grip strength persisted at genome-wide significance even after sensitivity analyses restricted to participants without any form of self-reported condition which might affect muscle mass or function. Whilst these clinical conditions represent the extreme phenotype of highly deleterious rare mutations in these genes, we demonstrate that proximal common variants are likely to underpin more subtle population-level variation in strength in healthy populations.

Finally, we found that common variation at *ACVR2B*, the principal receptor of myostatin and activin in skeletal muscle, is associated with population-level variation in grip strength. Ongoing clinical trials and development of pharmaceutical agents targeting this pathway have demonstrated their potential to reverse atrophy and improve physical functioning^46^, and our findings provide some level of genetic support for a role in muscular strength.

In conclusion, we identified 16 loci robustly implicated in grip strength and provide insight into the underlying biology of this important, widely studied, yet poorly characterized trait. MR analyses suggest no causal role for muscular strength in mortality, but do provide evidence for a causal role in fracture risk, highlighting the importance of interventions to improve muscle strength as a means to reduce fracture risk and resultant morbidities. Further genetic and functional work to characterize these loci will elucidate new pathways involved in the regulation of muscle strength and inform the development of drugs to tackle muscle wasting and weakness.

## Methods

### Study cohorts

Stage one (discovery) analyses comprised participants drawn from the UK Biobank (UKB) study^17^, a large population-based cohort of middle and older-aged (40–69 years) British residents recruited from UK National Health Service (NHS) primary care registers between 2006 and 2010. In total, 503,325 participants were enrolled, and attended an initial assessment visit at one of 22 study centres located throughout England, Scotland and Wales, during which a comprehensive catalogue of anthropometric, lifestyle and behavioural exposures were assessed, and biological samples were attained. All participants provided informed consent. UKB gained ethical approval from the National Research Ethics Committee (North West) and was conducted in full compliance with principles of the World Medical Association Declaration of Helsinki.

Independent lead variants from stage one were followed-up (stage two analyses) in an independent sample of up to 53,145 white European individuals drawn from the Cohorts for Heart and Aging Research in Genomic Epidemiology (CHARGE) consortium^16^ and an additional seven collaborating studies, which had assessed maximal isometric hand grip strength by dynamometry. Details of all stage two cohorts (including the constituent cohorts of CHARGE) are provided in the Supplementary Note. Further descriptive details of the seven additional cohorts are provided in Supplementary Table 1. Descriptive details of CHARGE Cohorts have been published in detail elsewhere^16^.

### Assessment of grip strength and covariates

Hand grip strength at baseline in UKB was measured isometrically using a calibrated Jamar J00105 hydraulic hand dynamometer (Lafayette Instrument Company, IN, USA) adjusted to the individual’s hand size. With the participant seated, one measurement was taken per hand, and maximal grip strength taken as the higher of the two readings. Body mass was assessed using a BC418MA Body Composition Analyser (Tanita Europe BV, Amsterdam, The Netherlands), with the participant dressed in light clothing. Standing height was measured on a rigid stadiometer (Seca, Birmingham, UK). Phenotyping details for each of the stage two cohorts are detailed in Supplementary Table 1.

### Genotyping and imputation

Our stage one analyses use data from UKB’s imputed interim genotyping release (May 2015), restricted to biallelic SNVs with MAF ≥0.1%. Genotyping and imputation were conducted by UKB using a centralized pipeline, for which detailed protocols are available (see URLs). Briefly, UKB extracted DNA from EDTA buffy coat, before shipping to Affymetrix (Santa Clara, CA, USA) for centralized genotyping. Samples were genotyped at >800,000 loci on two custom-designed arrays with 95% common content, designed to optimize quality and quantity of genome-wide imputation: the UK Biobank Axiom array, and the UK BiLEVE Axiom Array. After restriction to biallelic SNVs with MAF≥1% and additional sample genotyping QC, a subset of 641,081 autosomal SNVs from 152,256 samples were available for imputation. SNVs were pre-phased using SHAPEIT3 software and imputed (using a modified version of IMPUTE2 software) to a merged reference panel containing haplotypes from the UK10K Consortium combined with the 1000 Genomes Project reference (see URLs). This approach has previously been shown to provide a high-quality imputation reference in populations of mixed ancestry^61^. Genotyping and imputation details of stage two cohorts are detailed in Supplementary Table 1.

### 17q21.31 Haplotype imputation and analyses

Nine structural haplotypes previously reported at 17q21.31 were imputed according to previous work^33^,^34^,^35^. Imputation was based on a haplotype reference panel^34^, which uniquely coded each structural haplotype using a combination of twelve surrogate, virtual binary markers. In addition, the file contained 6,302 flanking variant haplotypes. IMPUTE v2.3.2 was used to impute the genotypes of the surrogate markers against the reference panel. The panel contained 284 genotyped variants within the reference region, pre-phased with SHAPEIT v2.837. Variants within the copy-number variable region, or with MAF<0.01/Hardy-Weinberg Equilibrium *P*<1 × 10^−6^ were excluded. Surrogate markers were subsequently decoded into the corresponding nine structural haplotypes for analysis. Association of haplotypes (inverted versus non-inverted, continuous structural variant [*α*/*β*/*γ*] copy number, and 9 common haplotypes as a categorical exposure) with grip strength was modelled using linear regression adjusted for age, sex, height (m), BMI (kg m^−2^) and UKB genotype chip in up to 111,860 unrelated genetic white Europeans defined centrally by UKB (see URLs).

### Heritability estimation

In UKB, variance component analyses were performed in the subset of individuals of ‘white British’ genetic ancestry using Restricted Estimate Maximum Likelihood (REML) models in BOLT-LMM software (v2.2)^62^. Genetic variance was calculated on all quality controlled genotyped autosomal SNVs, adjusting for genotyping array and the top five genetically-determined principal components.

### Genome-wide association analyses of grip strength

142,035 UKB participants had imputed genetic data, grip strength and full covariate availability for genome-wide association analyses; all were of self-identified white ancestry, with the majority (94.6%) reporting as white British. Discovery analyses for maximal grip strength (*n*=142,035) were run using a Bayesian linear mixed model (LMM) adjusted for age (years), sex, height (m) and BMI (kg m^−2^), implemented in BOLT-LMM software (v2.2)^62^. Primary analyses assumed additive (per-allele) effect. Analyses were restricted to biallelic variants with MAF≥0.1% which had been directly typed, or imputed with imputation quality (IMPUTE2 info)≥0.4. LMMs offer a robust solution to handle unknown confounding (particularly that arising from sub-ethnic population stratification and cryptic relatedness) in genome-wide association studies, and confer increased power in large population-based cohorts^62^.

Independent loci from genome-wide discovery were defined as the 500 kb region flanking each lead variant reaching genome wide significance (*P*≤5 × 10^−8^). Independent lead variants (*n*=21) were followed-up in up to 53,145 individuals. In cases where an index variant was not typed or imputed at sufficient quality, appropriate proxies were defined as the variant with the next-lowest *P*-value within 500 kb of the index (Supplementary Table 15). Each stage two cohort accounted for population structure according to its usual practice. Full details of the analytical approach and model specification of each replication cohort are summarized in Supplementary Table 1. Stage one and stage two results for each of the 21 variants were combined by inverse variance-weighted fixed-effect meta-analysis using METAL. Sixteen loci reached *P*≤5 × 10^−8^ in combined meta-analysis and were considered to be associated with grip strength.

To examine local linkage disequilibrium structure of replicated loci, regional plots for each of the 16 replicated loci were generated in LocusZoom using LD reference values from the CEU panel of 1000G Phase I. To investigate possible independent signals at each of the 16 replicated loci, approximate conditional analyses were undertaken using Genome-Wide Complex Trait Analysis software (GCTA, Version 1.25.2).

### LD score regression

Using genome-wide summary statistics from our UKB phase one analyses, the recently-described LD Score Regression method described by Bulik-Sullivan and colleagues^63^ (implemented in LDSC software, v1.0.0) was used to (i) estimate genetic correlation between grip strength and other phenotypes, and (ii) derive tissue-specific partitioned heritability of grip strength, based on pre-calculated European LD Scores. To avoid confounding by imputation quality, all analyses were restricted to variants available in HapMap Phase III.

Genetic correlations. Using cross-trait LD Score regression, genome-wide genetic correlations of grip strength were calculated with CHD risk, lean mass index (LMI), fat mass index (FMI) and bone mineral density (BMD) measured at the forearm, femoral neck or lumbar spine. For CHD and BMD we used publicly available GWAS summary statistics form the CARDIoGRAMplusC4D^57^ and GEFOS^58^ consortia, respectively. To obtain genome-wide summary statistics for LMI and FMI, we conducted GWAS in up to 12 851 individuals drawn from the Fenland Study and EPIC-Norfolk. Details of these cohorts are provided in the Supplementary Note, and Supplementary Table 14. LMI and FMI (kg m^−2^) were defined as dual x-ray absorptiometry (DXA)-derived lean mass or fat mass, respectively, divided by the square of DXA-derived height (GE Lunar Prodigy processed using Lunar EnCORE v14.1, GE Healthcare). GWAS were conducted separately in each cohort running a linear mixed model using BOLT-LMM^62^. FMI analyses were adjusted for age and sex. Because of the sex specific distribution of the phenotype, LMI analyses were run sex stratified and adjusted for age. Results from both cohorts were combined by fixed-effect inverse variance-weighted meta-analysis using METAL.

Tissue-specific partitioned heritability: Partitioned heritability by LD Score Regression can identify whether certain cell types are enriched for functional gene categories which disproportionately contribute to the heritability of a phenotype^36^. In this way, over-represented effector tissues important in the aetiology of the phenotype can be identified. Partitioned heritability was run individually for each of the eight curated tissue classes distributed with LDSC, adjusting in each case for heritability explained by each functional category of variants across the genome (that is, in a non-tissue-specific manner). A Bonferroni-corrected log_10_ *P-*value accounting for eight tests was taken as indicative of statistical significance.

### Expression analyses

To explore the potential functional significance of grip strength variants in gene expression, and to prioritize functional genes falling within identified loci, we undertook a number of expression-based analyses. Initially, a look-up of all 16 replicated grip strength variants or their best proxy (*r*^2^>0.8) was conducted in skeletal muscle, transformed fibroblasts, nervous system and brain regions in the GTEx resource to identify eQTL associations (see URLs). Variants passing GTEx criteria for tissue-specific eQTL association, and in high LD (*r*^2^≥0.8) with the best eQTL for the transcript in question in the tissue of interest were considered significant eQTLs.

To supplement this variant-centric approach, we additionally took advantage of two new methods for integrating genome wide GWAS summary statistics with expression associations from independent studies: SMR^40^ and MetaXcan^39^. By utilizing established eQTL data sets as reference, these approaches are able to effectively model expected variation in the transcriptome of the GWAS sample based on variation in autosomal SNVs across the genome, and then test for independent associations between imputed transcript levels and the phenotype of interest. To test for associations of transcript abundance with grip strength in whole blood, we implemented SMR software using published whole blood eQTL data from Westra and colleagues^41^ as the reference panel. MetaXcan—an extension of the PrediXcan approach modified to use summary-level association statistics as input—analyses were used to explore further tissue-specific associations between modelled gene expression and grip strength. Tissue-specific expression prediction models generated from GTEx were downloaded from the PredictDB resource (see URLs) as transcriptome reference. We conservatively considered predicted expression of a gene to be associated with grip strength at a MetaXcan *P*-value≤2.52 × 10^−7^, taking each gene association in each tissue as an independent test for the purposes of Bonferroni correction.

### Gene set enrichment analyses

Genome-wide discovery results from the UKB cohort were tested for enrichment of pre-specified gene sets based on common functional annotation or known biological pathways in MAGENTA (v2.4)^64^. Enrichment was assessed in a hypothesis-free manner across gene sets drawn from six public databases of gene ontology, functional annotation and canonical/curated pathways: GO Terms, the Protein Analysis through Evolutionary Relationships (PANTHER) database, Ingenuity, the Kyoto Encyclopaedia of Genes and Genomes (KEGG), Biocarta and Reactome pathways (downloaded via the Molecular Signatures Database, MSigDB [see URLs], July 2011). Analyses were restricted to 3,216 gene sets with an effective size of ≥10 genes after filtering of proximal genes and genes not containing variants for analysis. Custom sets were also defined from literature review, incorporating genes with known function in pathways or processes relevant to muscle development and maintenance. Genes involved in signal transduction of myostatin/activin signalling *via* Activin A type II receptors (*ACVR2A* and *ACVR2B*) were defined from Han *et al*.^42^. Monogenic genes implicated in muscular dystrophies and myopathies were based on Kaplan & Hamroun (2014)^65^ with additional manual curation to include collagen IV-opathies, congenital myopathies and glycogen storage diseases which may present with a similar pattern of limb-girdle muscle weakness.

### Gene-based association tests

Genes involved in myostatin/activin signalling *via* activin type II receptors (*FST, MSTN, ACVR2A, ACVR2B*) and known atrophy effectors *TRIM63* and *FBXO32* were identified from literature^42^,^43^ and defined as candidate genes for grip strength based on their biological prior for an involvement in skeletal muscle trophism. Gene-based association tests were performed for each candidate using the Versatile Gene-based Association Study (VEGAS) algorithm^44^, calculating LD from HapMap Europeans. VEGAS was applied to the grip strength discovery-phase association results across the whole genome, restricting to directly typed and well-imputed variants (IMPUTE info>0.8).

### Muscle histology lookup

To identify whether grip strength variants were associated with elements of muscle histology, we looked-up each of the 16 replicated loci from combined analyses in a pre-existing GWAS of muscle histology parameters in a sample of 656 men from three independent cohorts of Swedish ancestry (see Supplementary Note for cohort details). Specifically, we investigated the linear (additive) association of each of the 16 lead SNVs with percentage of (i) type I fibres, (ii) type IIA fibres and (iii) type IIB fibres from muscle biopsy, as well as capillary density (calculated as the number of capillaries divided by the total number of fibres). Phenotypes were inverse-normalized prior to analysis. To better quantify power in this sample, formal power calculations were performed using Quanto (see URLs).

### Mendelian randomization analyses

We performed summary statistic Mendelian randomization (MR)^51^,^52^ to test whether genetically determined sex and growth hormone-related phenotypes were causally associated with grip strength. As primary analyses we performed inverse variance weighted summary statistics MR^52^. In addition, as sensitivity analyses for robust causal inference we tested for heterogeneity using Cochran’s Q test, ran MR-Egger^66^ to assess for pleiotropic effect, and additionally used a weighted median estimator and penalized weighted median estimator^67^. We used publicly available genome-wide association results for sex hormone binding globulin (SHBG)^53^, dehydroepiandrosterone sulphate (DHEA-S)^54^, fasting insulin^68^, insulin secretion^69^ and insulin-like growth factor-I (IGF-I)^55^. The variants included in the MRs are listed in Supplementary Table 16. Grip strength summary statistics were obtained from our stage one GWAS in UKB.

To infer causality in the association of grip strength with CHD, myocardial infarction (MI), fracture risk, BMD (forearm, lumbar spine, femoral neck), LMI and FMI, we ran summary statistic MR as described above using the 16 identified loci as instrumental variable for genetically-determined grip strength. For CHD, BMD, LMI and FMI we used the same GWAS summary statistics as we used to test genetic correlations (see above). In addition, we used publicly available MI summary statistics from the CARDIoGRAMplusC4D consortium^57^, fracture risk summary statistics from an ongoing analysis by the GEFOS consortium (Supplementary Note), and individual fracture risk data in EPIC-Norfolk. Where grip strength lead SNVs were not available in the outcome phenotype summary statistics, proxies were defined as the variant with the next-lowest *P*-value for association with grip strength within 500 kb of the index in stage one (UKB). All variants included in the analyses are detailed in Supplementary Table 18. Because EPIC-Norfolk was included in the LMI and FMI GWAS meta-analyses, and in the individual level data fracture risk analyses, we used the grip strength effect sizes obtained after exclusion of the EPIC-Norfolk study in the grip strength-LMI, grip strength-FMI and individual level grip strength-fracture risk MR analyses (Supplementary Table 17). On the individual level fracture risk data we ran a logistic regression model adjusted for age and sex. Summary statistics and individual level fracture risk MR results from GEFOS and EPIC-Norfolk were meta-analysed using fixed effects meta-analysis.

To test the causal relationship with all-cause mortality, we calculated a genetic grip strength risk score per individual in the EPIC-Norfolk study (*n* total=21,043, *n* cases=5,699 cases) based on the number of grip strength-increasing alleles weighted by the effect size from the combined phase one and follow-up analyses. We used effect sizes obtained by fixed-effect inverse variance-weighted meta-analysis of the phase one and two results, excluding EPIC-Norfolk, to generate weights that were independent of EPIC-Norfolk (Supplementary Table 17). The genetic risk score to mortality association was tested under a Cox proportional hazards model adjusted for age and sex. Proportional hazards were confirmed using standard technique.

We also sought to improve power by using parental lifespans in UKB (paternal: *n*_TOTAL_=133,123, *n*_DEATHS_=102,072; maternal: *n*_TOTAL_=138,096, *n*_DEATHS_=83,315), in line with previous work^56^. Parental lifespans and alive/dead status were regressed using Cox models on offspring genotype, in effect imputing parent genotype from offspring. The effects observed thus reflect the effect of offspring genotype on parental phenotype, and the expected allelic dosages in the parental generation are half the measured dosages in offspring. Effect estimates per parental allele are correspondingly twice that observed per offspring allele: results shown are the effect of one allele in parents on parents’ lifespan.

### Association of replicated loci with elite athletic status

Using data from four multi-ethnic cohorts of elite athletes, including elite Japanese athletes and controls (*N*_athletes_=54, *N*_controls_=406); elite African-American ((*N*_athletes_=79, *N*_controls_=391) and Jamaican sprint/power athletes (*N*_athletes_=88, *N*_controls_=87), and European athletes (*N*_athletes_=395, *N*_controls_=726) (Supplementary Note), we assessed the association of the 16 replicated grip strength index variants with odds of attaining elite athlete status, relative to age, sex and ethnically-matched controls, using conditional logistic regression (additive model). Analyses were performed separately in each cohort, and meta-analysed using METAL.

### Tests of model fit

To test for departure from additivity, we used a test of dominance deviation, including two terms for best guess genotypes: a term encoding the major homozygotes, heterozygotes and minor allele homozygotes as 0,1,2 and another coding them as 0,1,0, which tests whether the heterozygotes have mean trait values halfway between the homozygote groups and can detect a departure from additivity.

### Checks for allele selection by age

Given that observational grip strength is strongly predictive of mortality^8^, we ran two complementary analyses in UKB to ensure that strength-increasing alleles from combined stage one+two analyses were not under selection by age. Modelling each SNV as strength-increasing allele dosage, linear regression was used to assess the association of age with allele dosage (age as dependent variable). We then performed the inverse of this regression to gauge whether allele dosage was predicted by age (age as the independent variable). This approach has recently been applied to test for selection of variants by age in the Genetic Epidemiology Research on Aging (GERA) cohort^18^. Analyses were restricted to 112,337 unrelated white Europeans defined centrally by UKB, and adjusted for sex and genotyping chip.

### URLs

UK Biobank Genotyping and QC Documentation http://biobank.ctsu.ox.ac.uk/crystal/docs/genotyping_qc.pdf; UK Biobank Phasing and Imputation Protocol; http://biobank.ctsu.ox.ac.uk/crystal/docs/impute_ukb_v1.pdf; MSigDB; http://software.broadinstitute.org/gsea/msigdb; SMR; http://cnsgenomics.com/software/smr; PredictDB Database; http://predictdb.hakyimlab.org; Quanto; http://biostats.usc.edu/Quanto.html

### Data availability

Stage one data are from UK Biobank, and can be obtained upon application (ukbiobank.ac.uk). Access to underlying replication and follow-up data including histology and elite athletic performance cohorts may be limited by participant consent and data sharing agreements; requests should be directed in the first instance *via* the corresponding authors. Pre-defined gene sets (MSigDB), expression data (GTEx) and transcriptome models used by MetaXcan (PredictDB) and SMR methods are available from the listed URLs.

## Additional information

**How to cite this article:** Willems, S. M. *et al*. Large-scale GWAS identifies multiple loci for hand grip strength providing biological insights into muscular fitness. *Nat. Commun.* **8,** 16015 doi: 10.1038/ncomms16015 (2017).

**Publisher’s note:** Springer Nature remains neutral with regard to jurisdictional claims in published maps and institutional affiliations.

## Supplementary Material

Supplementary Information

Supplementary Data

## Figures and Tables

**Table 1:** Association of the sixteen loci reaching genome-wide significance in combined analyses.

				**Stage one (UKB)**†			**Stage two cohorts**			**Combined**			
**rsID**	Gene*	**All.**	**EAF**	**Effect**‡	**S.E.**	***P-*****value**	**Effect**‡	**S.E.**	***P-*****value**	**Effect**‡	**S.E.**	***P-*****value**	***N***
rs958685	*TGFA*	A/C	0.52	0.154	0.026	2.8 × 10^−9^	0.164	0.04	3.8 × 10^−5^	0.157	0.022	4.8 × 10^−13^	191,754
rs72979233	*POLD3*	A/G	0.76	0.210	0.03	3.7 × 10^−12^	0.112	0.041	5.8 × 10^−3^	0.175	0.024	5.0 × 10^−13^	192,490
rs11614333	*ERP27*	C/T	0.62	0.181	0.027	5.0 × 10^−11^	0.117	0.04	3.5 × 10^−3^	0.16	0.023	1.6 × 10^−12^	195,154
rs2288278	*HOXB3*	A/G	0.66	0.162	0.027	3.0 × 10^−9^	0.147	0.04	2.8 × 10^−4^	0.157	0.023	3.8 × 10^−12^	195,133
rs4926611	*GLIS1*	C/T	0.64	0.173	0.027	1.3 × 10^−10^	0.115	0.041	5.1 × 10^−3^	0.156	0.023	4.8 × 10^−12^	192,964
rs6687430	*PEX14*	G/A	0.46	0.15	0.026	7.6 × 10^−9^	0.124	0.04	1.7 × 10^−3^	0.142	0.022	5.6 × 10^−11^	195,176
rs10186876	*LRPPRC*	A/G	0.36	0.162	0.027	2.7 × 10^−9^	0.113	0.041	6.2 × 10^−3^	0.147	0.023	9.8 × 10^−11^	192,490
rs374532236	*MGMT*	T/C	0.38	0.157	0.027	5.5 × 10^−9^	0.121	0.042	4.2 × 10^−3^	0.147	0.023	1.1 × 10^−10^	189,701
rs10861798	*SYT1*	A/G	0.43	0.145	0.026	4.3 × 10^−8^	0.159	0.047	7.4 × 10^−4^	0.148	0.023	1.3 × 10^−10^	189,160
rs78325334	HLA	T/C	0.84	0.228	0.038	2.4 × 10^−9^	0.113	0.05	0.024	0.186	0.03	9.6 × 10^−10^	193,127
rs2273555	*GBF1*	A/G	0.61	0.153	0.027	9.1 × 10^−9^	0.096	0.041	0.019	0.136	0.022	1.1 × 10^−9^	191,754
rs80103986	*KANSL1*	A/T	0.81	0.201	0.033	1.8 × 10^−9^	0.098	0.052	0.059	0.171	0.028	1.2 × 10^−9^	193,090
rs2110927	*SLC8A1*	C/T	0.27	0.161	0.029	4.4 × 10^−8^	0.098	0.045	0.029	0.142	0.025	7.7 × 10^−9^	192,490
rs6565586	*ACTG1*	A/T	0.25	0.169	0.03	2.2 × 10^−8^	0.096	0.064	0.14	0.156	0.027	1.2 × 10^−8^	187,072
rs72762373	*DEC1*	A/G	0.03	0.424	0.078	4.9 × 10^−8^	0.359	0.255	0.16	0.418	0.074	1.8 × 10^−8^	152,162
rs34845616	*IGSF9B*	A/G	0.25	0.168	0.03	1.7 × 10^−8^	0.07	0.049	0.15	0.141	0.025	2.7 × 10^−8^	189,666

All, alleles (effect/other); EAF, effect allele frequency; HLA, HLA region; *N*, sample size; UKB, UK Biobank.

Results are sorted by combined stage one+stage two *P*-value.

^*^Nearest gene to the lead SNP.

^†^Stage one analyses include 142,035 participants.

^‡^Effect estimates are in kg per allele and correspond to the first allele shown.
