# Supplementary material for: Large-scale GWAS identifies multiple loci for hand grip strength providing biological insights into muscular fitness
Source: Nat Commun. 2017 Jul 12;8:16015. doi: 10.1038/ncomms16015 (PMC5510175; doi:10.1038/ncomms16015)
Supplement: Supplementary Information [file ncomms16015-s1.pdf]

File name: Supplementary Information
Description: Supplementary Figures and Notes.

File name: Supplementary Data
Description: Excel Workbook containing Supplementary Tables 1-18 as separate worksheets. Each table is self-contained and includes a legend and title within the worksheet.

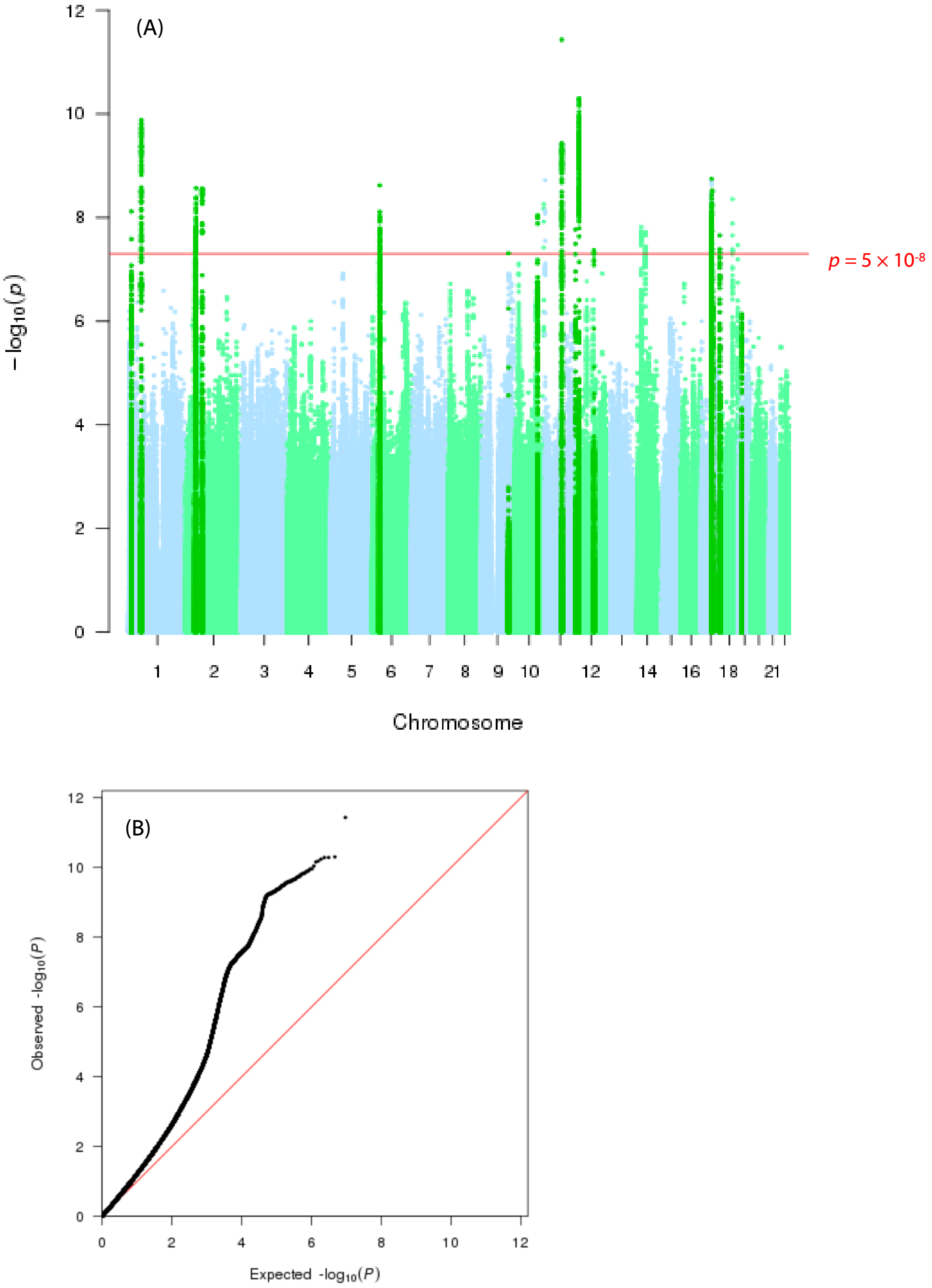

Supplementary Figure 1

(A) Manhattan plot of stage one association analyses in UK Biobank. Independent loci which retain genome-wide significance in the combined analysis are shown in bright green.

(B) QQ-plot of stage one analyses in UK Biobank. λGC = 1.31. LD score regression intercept = 1.02 (95% CI 0.998 – 1.042) , suggesting that apparent inflation is attributable to polygenicity of grip strength[1]

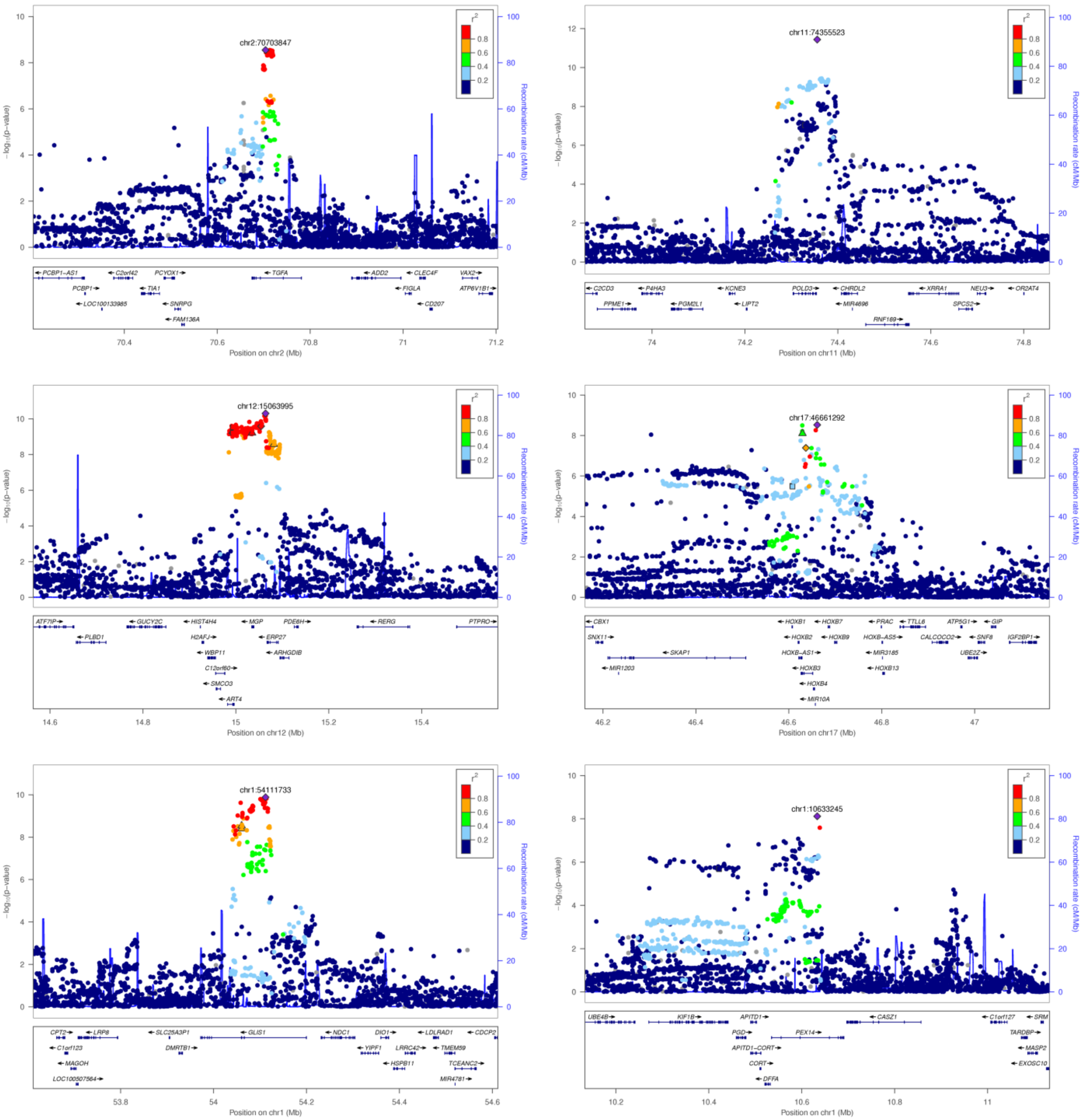

Supplementary Figure 2 (1 of 3)

Regional association plots of sixteen loci reaching genome-wide significance in combined stage one and stage two analyses. Outlined plot symbols denote a suggestive variant ($p<5\times10^{-6}$) with coding consequences according to Variant Effect Predictor: square = non-deleterious, inverted triangle = stop-gain, triangle = missense, diamond = splice region.

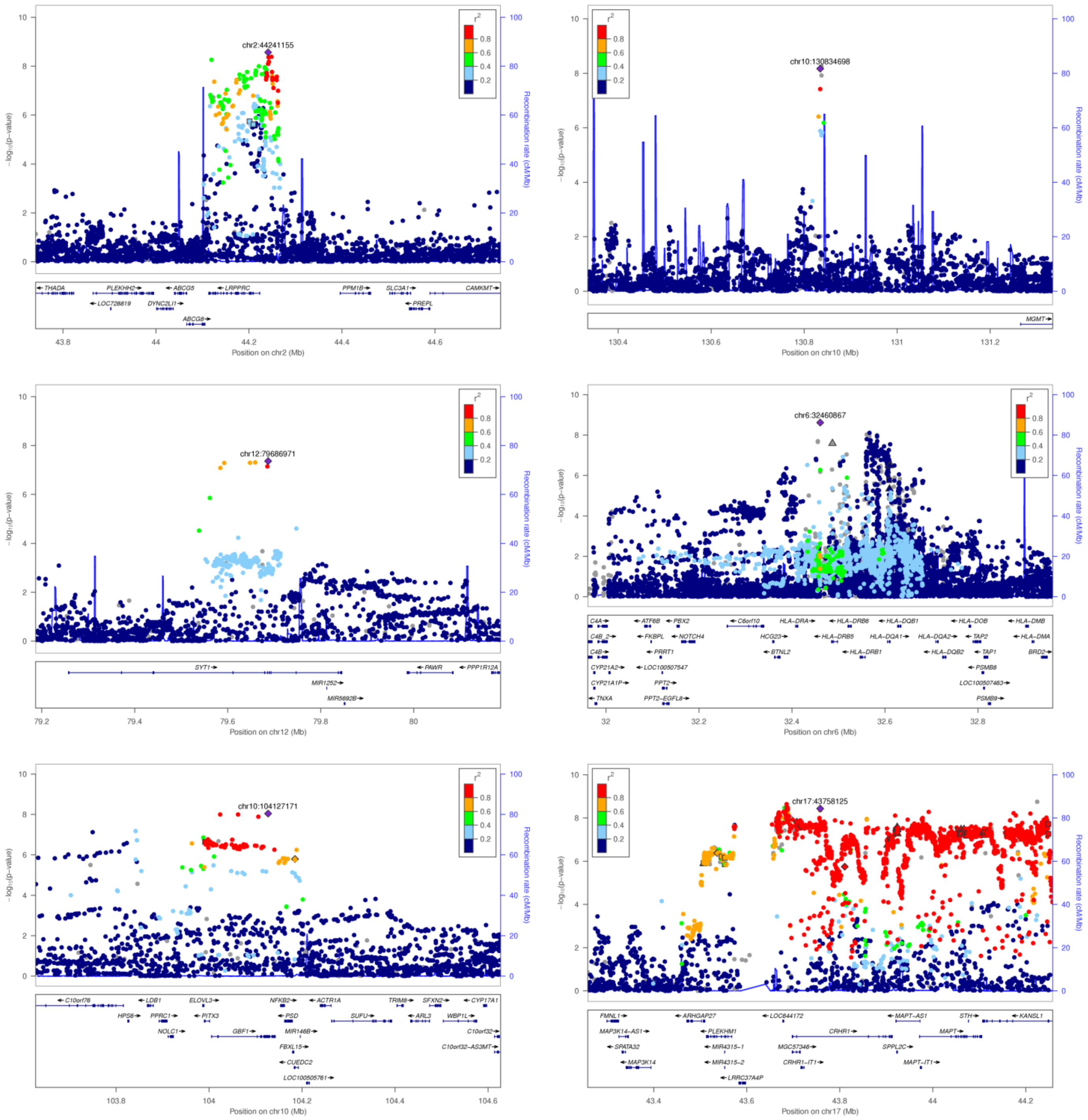

Supplementary Figure 2 (2 of 3)

Regional association plots of sixteen loci reaching genome-wide significance in combined stage one and stage two analyses. Outlined plot symbols denote a suggestive variant ($p<5\times10^{-6}$) with coding consequences according to Variant Effect Predictor: square = non-deleterious, inverted triangle = stop-gain, triangle = missense, diamond = splice region.

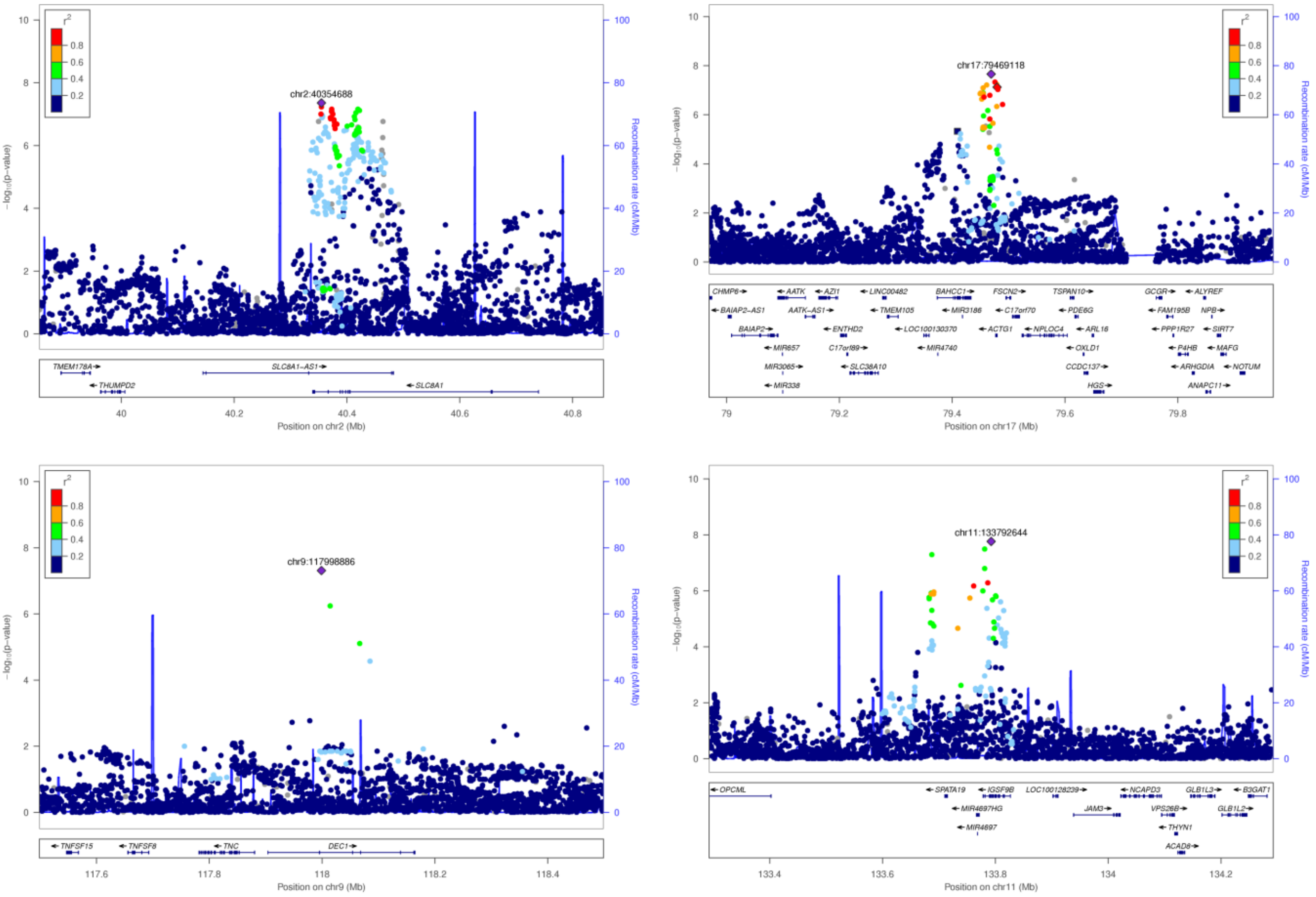

Supplementary Figure 2 (3 of 3)

Regional association plots of sixteen loci reaching genome-wide significance in combined stage one and stage two analyses. Outlined plot symbols denote a suggestive variant ($p<5\times10^{-6}$) with coding consequences according to Variant Effect Predictor: square = non-deleterious, inverted triangle = stop-gain, triangle = missense, diamond = splice region.

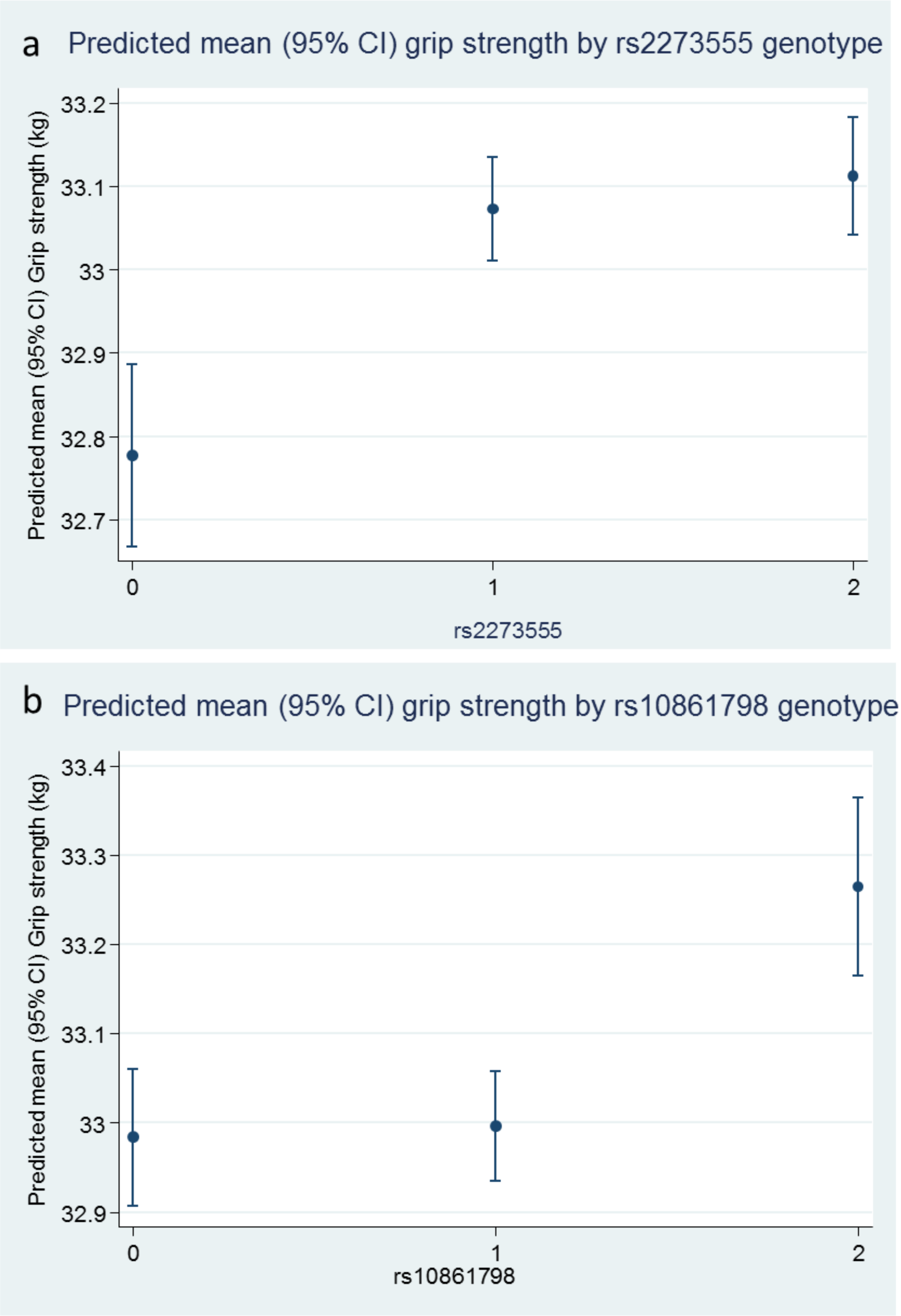

Supplementary Figure 3

Predicted mean grip strength by grip strength-increasing allele dosage at (a) rs2273555 (*GBF1*) and (b) rs10861798 (*SYT1*)

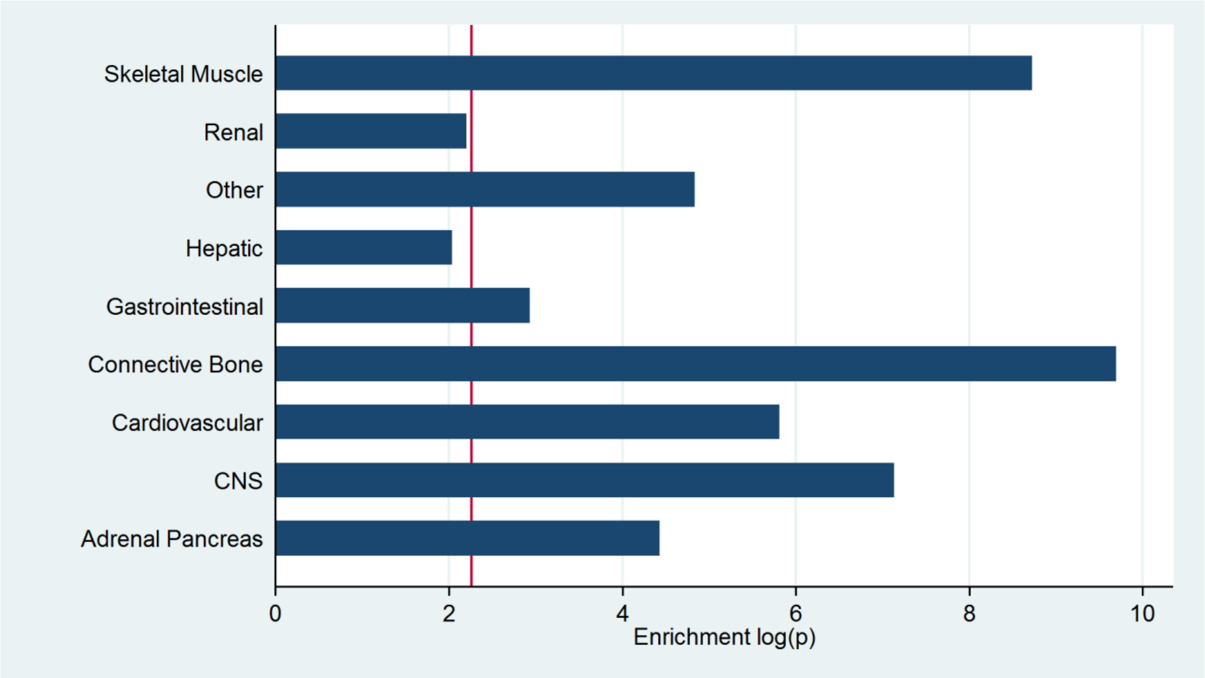

Supplementary Figure 4

Tissue-specific partitioned heritability enrichment of grip strength associations across the genome

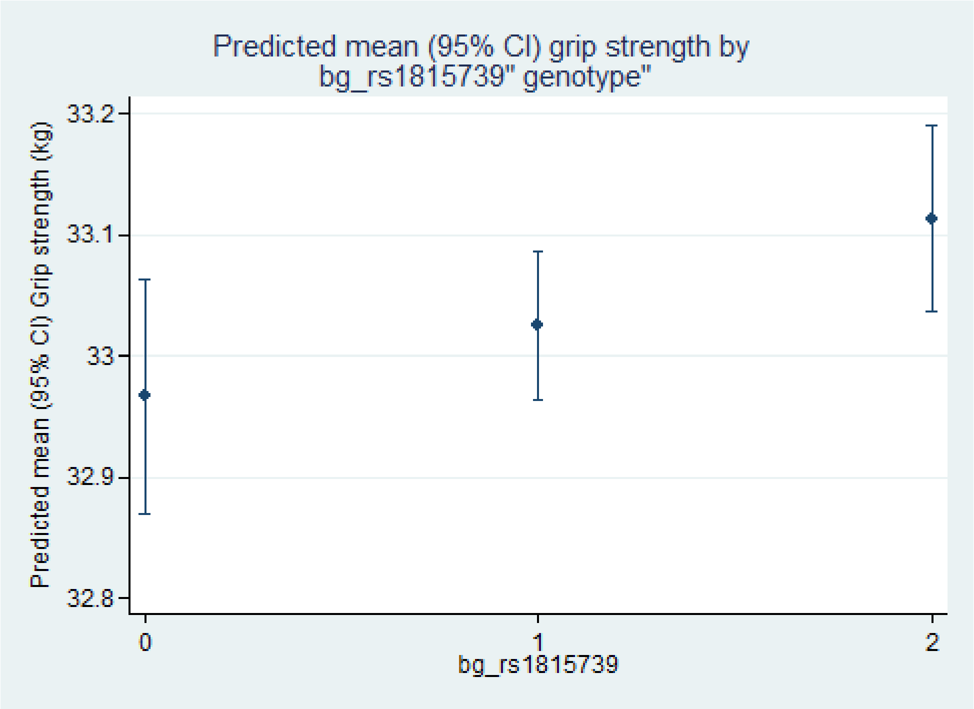

Supplementary Figure 5

Predicted mean grip strength by increasing dosage of the stop-gain allele (T) at rs1815739 (*R577X*)

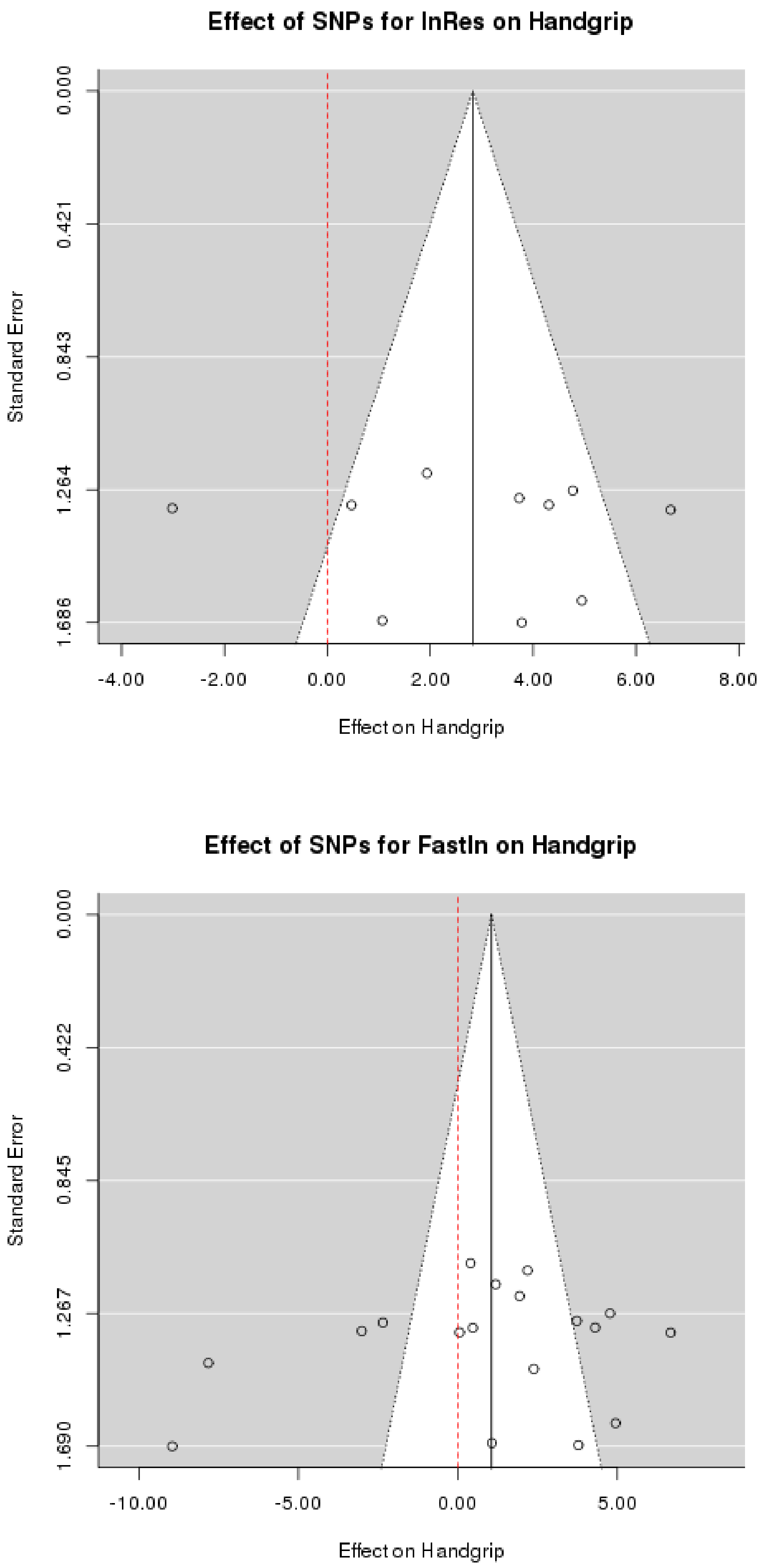

Supplementary Figure 6

Funnel plots showing heterogeneity in the effect of SNPs for insulin resistance and fasting insulin on grip strength

Stage II Cohorts

Informed consent was attained from participants in all contributing cohorts. Each contributing study was approved by a Local Institutional Review Board in accordance with each study's own procedures for gaining such approval. Where not stated in this document, full details of ethical approvals are included in the main reference provided for the study.

EPIC-Norfolk

Part of the international multi-centre European Prospective Investigation of Cancer (EPIC), EPIC-Norfolk (www.srl.cam.ac.uk/epic/) is an ongoing prospective population-based observational study of British adults aged 40-79 years at recruitment, enrolled between 1993-1997 by direct contact from collaborating NHS General Practice lists in Norwich and Norfolk, UK. The initial EPIC-Norfolk sample contains 25639 individuals who attended a baseline assessment upon recruitment, and have been contacted for follow-up assessments and repeat measures at intervals since. The data used in the present analyses is from the third health check (2004-2011), during which detailed anthropometric and lifestyle factors were characterised in participants, and blood drawn for biomarker analysis and DNA extraction [2]. Details of the genotyping process, quality control and imputation for EPIC-Norfolk are detailed in Supplementary Table 1. Written informed consent was supplied by all participants. Ethical approval for EPIC-Norfolk was granted by the Norfolk and Norwich Research Ethics Committee (now the East of England Research Ethics Committee).

The Fenland Study

The Fenland Study (www.mrc-epid.cam.ac.uk/research/studies/fenland/fenland-technical-summary/) is an ongoing, population-based cohort study (started in 2005) designed to investigate the association between genetic and lifestyle environmental factors and the risk of obesity, insulin sensitivity, hyperglycemia and related metabolic traits in men and women aged 30 to 55 years [3]. Potential volunteers were recruited from National Health Service (NHS) General Practice registrations in the Fenland, Ely and Cambridge areas of the Cambridgeshire Primary Care Trust, UK. Exclusion criteria for the study were: prevalent diabetes, pregnant and lactating women, terminal illness with prognosis less than one year from the date of contact, psychotic illness, or inability to walk unaided. Baseline phenotype assessment was undertaken during a dedicated assessment visit at MRC Epidemiology Unit Clinical Research Facilities in Ely, Wisbech or Cambridge. Participants attended after an overnight fast for a detailed clinical examination, and blood samples were collected. The Local Research Ethics Committee granted ethical approval for the study and all participants gave written informed consent.

Cohorts for Heart and Aging Research in Genomic Epidemiology (CHARGE) Consortium

Fourteen cohorts contributed to the CHARGE grip strength GWAS [4]:

AGES: The AGES–Reykjavik Study is a population-based study of older individuals from the 40-year long Reykjavik Study. Participants were aged between 66 and 96 years and were randomly recruited between 2002 and 2006 from surviving Reykjavik Study members[5]. Informed consent was obtained from all participants. Details of the investigations and local institutional review board approvals are described in the study's baseline article[5].

CHS: The CHS is a population-based cohort study of risk factors for CHD and stroke in adults ≥65 years conducted across four field centers[6]. The original predominantly Caucasian cohort of 5,201 persons was recruited in 1989-1990 from random samples of the Medicare eligibility lists; subsequently, an additional predominantly African-American cohort of 687 persons was enrolled for a total sample of 5,888. DNA was extracted from blood samples drawn on all participants at their baseline examination in 1989-90. In 2007-2008, genotyping was performed at the General Clinical Research Center's Phenotyping/Genotyping Laboratory at Cedars-Sinai using the Illumina 370CNV BeadChip system on 3980 CHS participants who were free of CVD at baseline, consented to genetic testing, and had DNA available for genotyping

FHS: The Framingham Heart Study was initiated in 1948 to study determinants of cardiovascular disease and other major illnesses. The Original Cohort included 5,209 men and women, aged 28-62 years at enrollment who have undergone routine biennial examinations [7]. In 1971, Offspring of the Original Cohort participants and Offspring spouses including 5,124 men and women, aged 5 to 70 years, were enrolled into the Framingham Offspring Study. Offspring participants have been examined approximately every 4 years. In the 1990s, DNA was obtained for genetic studies

from surviving Original Cohort and Offspring participants. All participants provided informed consent for all assessments through the Boston University Medical Center IRB.

Health-ABC: Between March 1997 and July 1998, 3,075 black and white men and women aged 70 to 80 were recruited to participate in the Health ABC Study; characteristics of the cohort have been described elsewhere [8]. Medicare beneficiary listings were used to recruit in metropolitan areas surrounding Pittsburgh, Pennsylvania, and Memphis, Tennessee. Eligibility criteria included having no difficulty walking one-quarter of a mile, climbing 10 steps, or performing activities of daily living (transferring, bathing, dressing, and eating); no history of active treatment for cancer in the prior 3 years; and no plans to move from the area within 3 years.

HRS: The Health and Retirement Study (HRS) is a longitudinal survey of a representative sample of Americans over the age of 50. The current sample is over 26,000 persons in 17,000 households. The study interviews respondents every two years about income and wealth, health and use of health services, work and retirement, and family connections [9]. DNA was extracted from saliva collected during a face-to-face interview in the respondents' homes. These data represent respondents who provided DNA samples and signed consent forms in 2006 and 2008.

InCHIANTI: The study participants consisted of men and women, aged 65 and older, who participated in the Invecchiare in Chianti, "Aging in the Chianti Area" (InCHIANTI) study, conducted in two small towns in Tuscany, Italy. The rationale, design, and data collection have been described elsewhere, and the main outcome of this longitudinal study is mobility disability [10]. Briefly, in August 1998, 1270 people aged 65 years and older were randomly selected from the population registry of Greve in Chianti (pop. 11,709) and Bagno a Ripoli (pop. 4,704), and of 1,256 eligible subjects, 1,155 (90.1%) agreed to participate. Participants received an extensive description of the study and participated after written, informed consent. The study protocol complied with the Declaration of Helsinki and was approved by the Italian National Institute of Research and Care on Aging Ethical Committee and by the Institutional Review Board of the Johns Hopkins University School of Medicine.

LBC: The Lothian Birth Cohort 1921 (LBC1921) cohort consists of 550 relatively healthy individuals, 316 females and 234 males, assessed on cognitive and medical traits at 79 years of age. They were born in 1921, most took part in the Scottish Mental Survey of 1932, and almost all lived independently in the Lothian region (Edinburgh City and surrounding area) in Scotland. When tested, the sample had a mean age of 79.1 years (SD = 0.6). A full description of participant recruitment and testing can be found elsewhere [11]. Ethics permission for the study was obtained from the Lothian Research Ethics Committee (LREC/1998/4/183). The research was carried out in compliance with the Helsinki Declaration. All subjects gave written, informed consent.

The Lothian Birth Cohort 1936 (LBC1936) consists of 1,091 relatively healthy individuals assessed on cognitive and medical traits at 70 years of age. They were born in 1936, most took part in the Scottish Mental Survey of 1947, and almost all lived independently in the Lothian region of Scotland. The sample of 548 men and 543 women had a mean age 69.6 years (SD = 0.8). A full description of participant recruitment and testing can be found elsewhere [12]. Ethics permission for the study was obtained from the Multi-Centre Research Ethics Committee for Scotland (MREC/01/0/56) and from Lothian Research Ethics Committee (LBC1936: LREC/2003/2/29). The research was carried out in compliance with the Helsinki Declaration. All subjects gave written, informed consent.

MAP: The Memory and Aging Project (MAP) is a longitudinal clinical-pathologic cohort study of aging. The study enrolls older adults without known dementia who agree to an annual assessment of risk factors, blood donation and a structured clinical evaluation [13]. All participants sign an Anatomical Gift Act and agree to the donation of brain, the entire spinal cord and selected nerve and muscles at the time of death. Study participants are primarily recruited from retirement communities throughout northeastern Illinois. Since October 1997, about 1,650 participants have completed their baseline evaluation. The follow-up rate of survivors exceeds 90%, and the autopsy rate exceeds 80%. The Religious Order Study (ROS) is a longitudinal clinical-pathologic cohort study of aging. The study enrolls older adults without known dementia who agree to an annual assessment of risk factors, blood donation and a detailed evaluation [14]. All participants sign an Anatomical Gift Act and agree to donation of brain at the time of death. Study participants are primarily Catholic priests, nuns and brothers from about 40 groups in 12 states. Since January 1994, over 1,100 participants completed their baseline evaluation. Both the follow-up rate among survivors and the autopsy rate exceed 90%.

MrOS: The Osteoporotic Fractures in Men Study (MrOS) is a multi-center prospective, longitudinal, observational study of risk factors for vertebral and all non-vertebral fractures in older men, and of the sequelae of fractures in men [15,16]. The original specific aims of the study include: (1) to define the skeletal determinants of fracture risk in older men, (2) to define lifestyle and medical factors related to fracture risk, (3) to establish the contribution of fall frequency to fracture risk in older men, (4) to determine to what extent androgen and oestrogen concentrations influence fracture risk, (5) to examine the effects of fractures on quality of life, (6) to identify sex differences in the predictors and outcomes of fracture, (7) to collect and store serum, urine and DNA for future analyses as directed by emerging evidence in the fields of aging and skeletal health, and (8) define the extent to which bone mass/fracture risk and prostate diseases are linked. From March 2000 to April 2002, 5994 community dwelling ambulatory men aged 65 years or older were recruited from six communities in the United States (Birmingham, AL; Minneapolis, MN; Palo Alto, CA; Monongahela Valley near Pittsburgh, PA; Portland, OR; and San Diego, CA). Inclusion criteria were designed to provide a study cohort that is representative of the broad population of older men. The MrOS inclusion criteria were: (1) ability to walk without the assistance of another, (2) absence of bilateral hip replacements, (3) ability to provide self-reported data, (4) residence near a clinical site for the duration of the study, (5) absence of a medical condition that (in the judgment of the investigator) would result in imminent death, (6) ability to understand and sign an informed consent, and (7) 65 years or older. To qualify as an enrollee, the participant had to provide written informed consent, complete the self-administered questionnaire (SAQ), attend the clinic visit, and complete at least the anthropometric, DEXA, and vertebral X-ray procedures. The institutional review board at each center approved the study protocol, and written informed consent was obtained from all the participants.

SHIP: The Study of Health in Pomerania (SHIP) is a population based in West Pomerania, the north-east area of Germany [17,18]. A sample from the population aged 20 to 79 years was drawn from population registries. First, the three cities of the region (with 17,076 to 65,977 inhabitants) and the 12 towns (with 1,516 to 3,044 inhabitants) were selected, and then 17 out of 97 smaller towns (with less than 1,500 inhabitants), were drawn at random. Second, from each of the selected communities, subjects were drawn at random, proportional to the population size of each community and stratified by age and gender. Only individuals with German citizenship and main residency in the study area were included. Finally, 7,008 subjects were sampled, with 292 persons of each gender in each of the twelve five-year age strata. In order to minimize drop-outs by migration or death, subjects were selected in two waves. The net sample (without migrated or deceased persons) comprised 6,267 eligible subjects. Selected persons received a maximum of three written invitations. In case of non-response, letters were followed by a phone call or by home visits if contact by phone was not possible. The SHIP population finally comprised 4,308 participants (corresponding to a final response of 68.7%). This study includes data of 320 individuals with complete GWAS data who participated in the 10-year follow-up examinations (SHIP-2).

SOF: The Study of Osteoporotic Fractures (SOF) is a prospective multicenter study of risk factors for vertebral and non-vertebral fractures [19]. From 1986 to 1987, 9704 community dwelling women aged 65 years or older were recruited from population-based listings in four U.S. areas: Baltimore, Maryland; Minneapolis, Minnesota; Portland, Oregon; and the Monongahela Valley, Pennsylvania. The SOF participants were followed up every four months by postcard or telephone to ascertain the occurrence of falls, fractures and changes in address. The SOF inclusion criteria were: 1) 65 years or older, (2) ability to walk without the assistance of another, (3) absence of bilateral hip replacements, (4) ability to provide self-reported data, (5) residence near a clinical site for the duration of the study, (6) absence of a medical condition that (in the judgment of the investigator) would result in imminent death, and (7) ability to understand and sign an informed consent. To qualify as an enrollee, the participant had to provide written informed consent, complete the self-administered questionnaire (SAQ), attend the clinic visit, and complete at least the anthropometric measures. The institutional review board at each center approved the study protocol, and written informed consent was obtained from all the participants.

TASCOG: TASCOG is a study of cerebrovascular mechanisms underlying gait, balance and cognition in a population-based sample of Tasmanian people aged at least 60 years. Individuals aged 60–86 years (n = 395) living in Southern Tasmania, Australia, were randomly selected from the electoral roll between 2006 and 2008 to participate in the study. Individuals were excluded if they lived in a nursing home, had a contraindication for magnetic resonance scanning (MRI) or were unable to walk without a gait aid18. Participants underwent brain MRI scans and genotyping. DNA was extracted from peripheral blood samples by proteinase K digestion following cell lysis, then phenol-chloroform purification. DNA was genotyped using Illumina Hap370CNV chips at the University of Queensland Diamantina Institute, Princess Alexandra Hospital, Brisbane, Australia, for 370 participants, and call rates were greater than 97% for all samples.

Genotypes for 22 individuals were excluded, either because they were closely related to other individuals, they were outliers in a population ancestry analysis or their sex predicted from genotypes did not match sex as recorded in the database. Among the 348 remaining participants with available genome-wide data, after exclusion of 2 participants with dementia, 3 with posterior circulation infarcts on MRI and 3 with insufficient MRI image quality, 340 individuals were available for the present analysis on hippocampal volume.

TUK: The TwinsUK registry now consists of about 12,000 monozygotic (MZ) and dizygotic (DZ) twins aged 18 to 103 years. About 83% of the registry is female (mean age of 55 years). The registry now contains 51% MZ and 49% DZ twins. Between 1992 and 2004, twins were invited for a full comprehensive visit and several project-led studies. More than 7,000 twins responded to some of the annual questionnaires and 5,725 attended a comprehensive visit. Apart from a lifelong lower weight in MZ twins of around 1 kg, all other age-matched characteristics of these volunteer twins were found not to differ from a singleton population-based cohort of British women [20]. Between April 2004 and May 2007, all the 6,740 active twins on the registry were invited for a 1-day clinical visit, of whom 3,725 twins attended and 1,299 twins posted their blood DNA samples via their general practitioners. The age of participants ranged between 18 and 82 years (mean 52.5 ± 13 years) and 3,299 of the clinic attendants (89%) were female.

Erasmus Rucphen Family Study (ERF)

The Erasmus Rucphen Family genetic isolate study (ERF) is a prospective family based study located in the southwestern Netherlands [21]. This young genetic isolate was founded in the mid-eighteenth century and minimal immigration and marriages occurred between surrounding settlements due to social and religious reasons. The ERF population includes 3,465 individuals that are living descendants of 22 couples with at least six children baptized. The study protocol was approved by the medical ethics board of the Erasmus MC Rotterdam, the Netherlands. The baseline demographic data and measurements of the ERF participants were collected around 2002 to 2006. All the participants filled out questionnaires on socio-demographics, disease and medical history and lifestyle factors, and were invited to the research center for an interview and blood collection for biochemistry and physical examinations. In the main follow-up of the study by May, 2016 (follow up from 9 to 14 years), 1,940 baseline participants' records were scanned in the general practitioner's database.

Health 2006 and 2008 Cohorts

The Health2006 study is a general population-based study (started in 2006) designed to investigate lifestyle-related chronic diseases such as coronary heart disease, diabetes, musculoskeletal disorders, asthma, allergy, chronic lung diseases and mental disorders in men and women aged 18 to 69 years. The Health2008 study (started in 2008) is an extension of the Health2006 study using essentially similar collection methods, investigating men and women aged 30 to 60 years, living in the same municipalities as participants from Health2006. A random sample of potential volunteers was obtained from the Danish Civil Registration system. All participants were Danish adults living in 11 municipalities in the south-western part of the greater Copenhagen area, Denmark. Pregnant women were excluded. All participants had measurements done at the Research Centre for Prevention and Health at the Copenhagen University Hospital in Glostrup. Participants were asked to be fasting at the day of examination for a detailed clinical examination when blood samples were collected. Written informed consent was obtained from all participants and the study was approved by the Ethical Committee of Copenhagen County and the Danish Data Protection Agency. The study is registered at www.clinical.trials.com.

Hunter Community Study

The HCS [22] is a population-based study comprised of middle-aged to older adults aged 55-85 years from Newcastle, Australia. Voting registration in Australia is compulsory and participants were recruited randomly from the electoral roll. Potential participants were excluded if they could not speak English and/or if they lived in an aged-care facility. The final sample included 3207 participants. Ethics approval was obtained from the Hunter New England Local Health District and University of Newcastle Human Research Ethics Committees and written informed consent was obtained from all participants. Comprehensive data was collected including samples for genetic analyses.

Sydney Memory & Ageing Study

The Sydney MAS [23] is a longitudinal population-based study, which recruited community-dwelling adults aged 70-90 years at baseline from Sydney. In Australia voting registration is compulsory and participants were recruited randomly from the electoral roll. Exclusion criteria included a diagnosis of dementia,

schizophrenia or bipolar disorder and medical or psychological conditions that would prevent them from completing assessments. The final sample was comprised of 1,037 participants at baseline. Ethics approval for Sydney MAS was granted by the Human Research Ethics Committees of the University of New South Wales and the South Eastern Sydney and Illawarra Area Health Service. Written informed consent was provided by all participants. A wide range of data was collected including samples donated for genetic analyses.

TwinsUK

In addition to the TUK sample contained within CHARGE, Twins UK contributed 3009 additional genotyped grip strength samples that were not included in the pre-existing CHARGE GWAS discussed above. The TwinsUK cohort (www.twinsuk.ac.uk) is an adult twin British registry shown to be representative of singleton populations and the United Kingdom population [20]. The mean age of the 3009 individuals was 52.5 (±10.9) years. Ethical approval was obtained from the Guy's and St. Thomas' Hospital Ethics Committee. Written informed consent was obtained from every participant to the study.

Athlete case-control studies

European Athletes

The European Athlete study consists of white European sprint and power athletes (n=395) and approximately geographically-matched white European controls (n=726). Participant DNA was whole genome amplified and normalised before SNP-based genotyping at each of the sixteen grip strength SNPs identified in combined analyses (Sequenom). Genotyping was conducted at the Australian Genome Research Facility (AGRF). 12 SNPs passed quality control (HWE $p \geq 0.05$) and genotype associations between cases and controls were assessed using conditional logistic regression with country as a covariate (conducted in R). All participants supplied written informed consent, which was approved by each respective cohort's institution. This includes approval by the institutional review boards of the the Children's Hospital at Westmead (2003/086), The Royal Children's Hospital Human Research Ethics Committee (35172), the Pomeranian Medical University Ethics Committee, Universidad Pablo Olavide, The Lithuanian National Committee of Biomedical Ethics, Aristotle University of Thessaloniki Research Committee, The Ethics Committee of the University of Cagliari, Ghent University Hospital, Belgium and the Nottingham Trent University Ethical Review Committee.

Japanese Athletes

The Japanese cohort is comprised of 54 international sprint/power track and field athletes and 406 geographically matched controls. Total DNA was isolated from venous blood and saliva by QIAamp DNA Blood Maxi Kit (QIAGEN, Hilden, Germany) and Oragene·DNA (DNA genotek, Ontario, Canada), respectively. DNA Samples from all subjects were genotyped on the HumanOmniExpress BeadChip (Illumina). Ten of the sixteen replicated SNVs associated with grip strength in the present study were available in this Japanese population either directly or by proxy ($r^2 \geq 0.8$). Genotyping quality of each polymorphism was checked by visually in GenomeStudio (Illumina). This study is part of the Japanese Human Athlome Project, focusing on the study of genes associated with physical performance and its related phenotypes [24].
Written consent was obtained from each subject, and the study was approved by the Ethics Committees of the Juntendo University, National Institute of Health and Nutrition and Japan Institute of Sports Sciences.

Jamaican and US Athletes

This cohort is comprised of high-level black athletes (n=167) and geographically matched controls (n=478). Samples were genotyped using the HumanOmniExpress or Omni1-Quad BeadChips (Illumina). Standard GWAS quality control was applied to the genotype data. Genetic associations were evaluated by logistic regression with adjustment for population structure. Genotype imputation with IMPUTE2 using the 1000G phase 3 reference panels was then conducted to increase power of GWA scans. This investigation aims to understand the influence of common genetic variants on performance through the use of athletic populations enriched with high-level competitors. All participants supplied written informed consent, which was approved by the UHWI/UWI/FMS Ethics Committee, University of West Indies, Jamaica and participating institutions in the United States.

GEFOS Consortium

The GEFOS consortium is an international collaboration of investigators dedicated to identify the genetic determinants of osteoporosis (http://www.gefos.org/). The original any-type of fracture GWAS meta-analysis comprised 24 GWA studies (20,439 cases, 78,843 controls) from populations across North America, Europe, East Asia and Australia, with a variety of epidemiological designs and patient characteristics. Cases were individuals (>18 years) with fractures confirmed by medical, radiological or questionnaire reports.

When possible, fractures of the fingers, toes and skull were excluded from analyses. GWAS genotyping was done by each study following standard manufacturer protocols followed by imputation to ~2.5 million SNPs from HapMap Phase II.

Muscle Histology Cohort

The muscle histology study was performed on 656 men from three independent cohorts of Swedish ancestry. These cohorts included Uppsala Longitudinal Study of Adult Men (ULSAM, n=482) [25], Malmö Men (MM, n=128) [26] and Malmö Exercise Intervention (MEI, n=46) [27]. The ULSAM cohort included non-diabetic males of age 71.0 ± 0.64 years and body-mass index (BMI) 26.3 ± 3.4 kg/m^2. The MM cohort consists of non-obese males aged 65.9 ± 2.0 years and with BMI 26.4 ± 3.4 kg/m^2, including 50 individuals with diagnosed Type II diabetes (T2D). The MEI cohort consists of male subjects aged 37.7 ± 4.3 years, VO_{2MAX} 32.0 ± 5.0 ml/kg/min and BMI 28.0 ± 3.1kg/m^2 with (n=22) and without (n=24) a first degree family member with T2D. The primary phenotypes included in the GWA-analysis were percentage of (i) type I, (ii) type IIa, and (iii) Type IIx fibres, as well as capillary density, calculated as number of capillaries divided by total number of fibres. Phenotypes were inverse-normalised for analysis. Genotype data in each cohort were imputed up to 35 million variants from the 1000 Genomes "all ancestries" reference panel (March 2012). Prephasing of haplotypes and imputation were performed using ShapeIT and IMPUTE2, respectively. The association within each cohort was performed using SNPTEST frequentist score additive model test.

REFERENCES – SUPPLEMENTARY NOTE

1. Bulik-Sullivan, B. K. *et al.* LD Score regression distinguishes confounding from polygenicity in genome-wide association studies. *Nat. Genet.* **47,** 291–295 (2015).

2. Hayat, S. A. *et al.* Cohort profile: A prospective cohort study of objective physical and cognitive capability and visual health in an ageing population of men and women in Norfolk (EPIC-Norfolk 3). *Int. J. Epidemiol.* **43,** 1063–72 (2014).

3. De Lucia Rolfe, E. *et al.* Association between birth weight and visceral fat in adults. *Am. J. Clin. Nutr.* **92,** 347–52 (2010).

4. Matteini, A. M. *et al.* GWAS analysis of handgrip and lower body strength in older adults in the CHARGE consortium. *Aging Cell* 1–9 (2016). doi:10.1111/acel.12468

5. Harris, T. B. *et al.* Age, Gene/Environment Susceptibility-Reykjavik Study: multidisciplinary applied phenomics. *Am. J. Epidemiol.* **165,** 1076–87 (2007).

6. Fried, L. P. *et al.* The Cardiovascular Health Study: design and rationale. *Ann. Epidemiol.* **1,** 263–76 (1991).

7. DAWBER, T. R., MEADORS, G. F. & MOORE, F. E. Epidemiological approaches to heart disease: the Framingham Study. *Am. J. Public Health Nations. Health* **41,** 279–81 (1951).

8. Harris, T. B. *et al.* Waist circumference and sagittal diameter reflect total body fat better than visceral fat in older men and women. The Health, Aging and Body Composition Study. *Ann. N. Y. Acad. Sci.* **904,** 462–73 (2000).

9. Sonnega, A. *et al.* Cohort Profile: the Health and Retirement Study (HRS). *Int. J. Epidemiol.* **43,** 576–85 (2014).

10. Ferrucci, L. *et al.* Subsystems contributing to the decline in ability to walk: bridging the gap between epidemiology and geriatric practice in the InCHIANTI study. *J. Am. Geriatr. Soc.* **48,** 1618–25 (2000).

11. Deary, I. J., Whiteman, M. C., Starr, J. M., Whalley, L. J. & Fox, H. C. The Impact of Childhood Intelligence on Later Life: Following Up the Scottish Mental Surveys of 1932 and 1947. *J. Pers. Soc. Psychol.* **86,** 130–147 (2004).

12. Deary, I. J. *et al.* The Lothian Birth Cohort 1936: a study to examine influences on cognitive ageing from age 11 to age 70 and beyond. *BMC Geriatr.* **7,** 28 (2007).

13. Bennett, D. A. *et al.* The Rush Memory and Aging Project: study design and baseline characteristics of the study cohort. *Neuroepidemiology* **25,** 163–75 (2005).

14. Bennett, D. A., Schneider, J. A., Arvanitakis, Z. & Wilson, R. S. Overview and findings from the religious orders study. *Curr. Alzheimer Res.* **9,** 628–45 (2012).

15. Blank, J. B. *et al.* Overview of recruitment for the osteoporotic fractures in men study (MrOS). *Contemp. Clin. Trials* **26,** 557–68 (2005).

16. Orwoll, E. *et al.* Design and baseline characteristics of the osteoporotic fractures in men (MrOS) study--a large observational study of the determinants of fracture in older men. *Contemp. Clin. Trials* **26,** 569–85 (2005).

17. John, U. *et al.* Study of Health In Pomerania (SHIP): a health examination survey in an east German region: objectives and design. *Soz. Praventivmed.* **46,** 186–94 (2001).

18. Völzke, H. *et al.* Cohort profile: the study of health in Pomerania. *Int. J. Epidemiol.* **40,** 294–307 (2011).

19. Cummings, S. R. *et al.* Appendicular bone density and age predict hip fracture in women. The Study of Osteoporotic Fractures Research Group. *JAMA* **263,** 665–8 (1990).

20. Moayyeri, A., Hammond, C. J., Hart, D. J. & Spector, T. D. The UK Adult Twin Registry (TwinsUK Resource). *Twin Res. Hum. Genet.* **16,** 144–9 (2013).

21. Henneman, P. *et al.* Prevalence and heritability of the metabolic syndrome and its individual components in a Dutch isolate: the Erasmus Rucphen Family study. *J. Med. Genet.* **45,** 572–7 (2008).

22. McEvoy, M. *et al.* Cohort profile: The Hunter Community Study. *Int. J. Epidemiol.* **39,** 1452–63 (2010).

23. Sachdev, P. S. *et al.* The Sydney Memory and Ageing Study (MAS): methodology and baseline medical and neuropsychiatric characteristics of an elderly epidemiological non-demented cohort of Australians aged 70-90 years. *Int. Psychogeriatr.* **22,** 1248–64 (2010).

24. Pitsiladis, Y. P. *et al.* Athlome Project Consortium: a concerted effort to discover genomic and other 'omic' markers of athletic performance. *Physiol. Genomics* **48,** 183–90 (2016).

25. Hedstrand, H. A study of middle-aged men with particular reference to risk factors for cardiovascular disease. *Ups. J. Med. Sci. Suppl.* **19,** 1–61 (1975).

26. Mootha, V. K. *et al.* PGC-1alpha-responsive genes involved in oxidative phosphorylation are coordinately downregulated in human diabetes. *Nat. Genet.* **34,** 267–73 (2003).

27. Elgzyri, T. *et al.* First-degree relatives of type 2 diabetic patients have reduced expression of genes involved in fatty acid metabolism in skeletal muscle. *J. Clin. Endocrinol. Metab.* **97,** E1332–7 (2012).
